# Supplementary material for: Scalable synthesis of phosphorescent SiO2 nanospheres and their use for angle-dependent and thermoresponsive photonic gels with multimode luminescence
Source: Nat Commun. 2025 Jul 18;16:6640. doi: 10.1038/s41467-025-61967-9 (PMC12274461; doi:10.1038/s41467-025-61967-9)
Supplement: Supplementary file 1 — Supplementary Information [file 41467_2025_61967_MOESM1_ESM.pdf]

Supplementary information

Scalable synthesis of phosphorescent SiO_2 nanospheres and their use for angle-dependent and thermoresponsive photonic gels with multimode luminescence

Changxing Wang, Yayun Ning, Yifan Yue, Guoli Du, Yuechi Xie, Jianing Li, Nazia Bibi, Xiaoxiang Wen, Jianing Li, Sen Yang,* Xuegang Lu*

School of Physics, MOE Key Laboratory for Nonequilibrium Synthesis and Modulation of Condensed Matter, Shaanxi Province Key Laboratory of Advanced Functional Materials and Mesoscopic Physics, Xi'an Jiaotong University, Xi'an 710049, China.

E-mail: xglu@mail.xjtu.edu.cn; yangsen@mail.xjtu.edu.cn

Table of Contents

Supplementary Fig. 1 Diameter distribution of RTP SiO_2 NPs

Supplementary Fig. 2 Morphology and diameter of uncalcined glucose doped SiO_2 NPs

Supplementary Fig. 3 Distribution of chemical elements of pure SiO_2 NPs

Supplementary Fig. 4 The morphology and diameter distribution of RTP SiO_2 NPs with different particle sizes

Supplementary Fig. 5 Fluorescence excitation dependence of RTP SiO_2 NPs

Supplementary Fig. 6 Afterglow photographs of RTP SiO_2 NPs powders

Supplementary Fig. 7 Temperature dependence of afterglow properties of RTP SiO_2 NPs

Supplementary Fig. 8 Photos of RTP SiO_2 NPs in various environments

Supplementary Fig. 9 Systematic study on the optical stability and mechanism of RTP SiO_2 NPs

Supplementary Fig. 10 Morphological characterization of RTP SiO_2 NPs after self-assembly

Supplementary Fig. 11 Optical characterization of PCs constructed from RTP SiO_2 NPs with different particle sizes

Supplementary Fig. 12 Photographs of three optical morphologies of multimode PCs
Supplementary Fig. 13 Angle-dependent reflection spectra of multimode PCs
Supplementary Fig. 14 FL and RTP characteristics of multimode PCs assembled with different particle sizes of RTP SiO_2 NPs
Supplementary Fig. 15 Afterglow photos of multimode PCs
Supplementary Fig. 16 Morphology and particle size distribution of CDs generated in situ inside SiO_2 NPs
Supplementary Fig. 17 Calcination temperature dependent RTP characteristics of RTP SiO_2 NPs
Supplementary Fig. 18 Relationship between RTP lifetime of RTP SiO_2 NPs and calcination temperature
Supplementary Fig. 19 Relationship between FL lifetime of RTP SiO_2 NPs and calcination temperature
Supplementary Fig. 20 PLQY of RTP SiO_2 NPs
Supplementary Fig. 21 The degree of crystallization of in situ CDs in RTP SiO_2 NPs
Supplementary Fig. 22 FT-IR spectra of RTP SiO_2 NPs
Supplementary Fig. 23 High-resolution Si *2p* results of RTP SiO_2 NPs
Supplementary Fig. 24 High-resolution C *1s* results of RTP SiO_2 NPs
Supplementary Fig. 25 High-resolution O *1s* results of RTP SiO_2 NPs
Supplementary Fig. 26 Absorption characteristic of RTP SiO_2 NPs
Supplementary Fig. 27 Exploration of the mechanism of decreased luminescence efficiency of RTP SiO_2 NPs calcined at excessively high temperatures
Supplementary Fig. 28 Three CDs calculation models based on HR-TEM and chemical characterization
Supplementary Fig. 29 Quantum chemical calculations for three kinds of CDs models
Supplementary Fig. 30 Results of the validation of the universality of the method
Supplementary Fig. 31 Demonstration of large-scale preparation of RTP SiO_2 NPs
Supplementary Fig. 32 Physical microstructure characterization of multimode PC gels
Supplementary Fig. 33 The influence of PC gels structure on FL on RTP
Supplementary Fig. 34 Angle-dependent PBGs of R-PC gel
Supplementary Fig. 35 Multimode angle-dependent chromatic properties of disordered micro-structured R-PC gel
Supplementary Fig. 36 FL and RTP angle-dependent characteristics of PC gels
Supplementary Fig. 37 Angle-dependent PBGs of B-PC gel and G-PC gel

Supplementary Fig. 38 Multimode angle-dependent chromatic properties of G-PC gel
Supplementary Fig. 39 Multimode angle-dependent chromatic properties of B-PC gel
Supplementary Fig. 40 Optical behavior of the incident angle of excitation light on R-PC gel
Supplementary Fig. 41 Variation of RTP lifetime on the incident angle of excitation light
Supplementary Fig. 42 Thermal transition properties of transparent states and scattering states of R-PC gel
Supplementary Fig. 43 Enhanced behavior of UV excited light in the scattered state
Supplementary Fig. 44 FL Lifetime of R-PC gel in refractive index matched state and mismatched state
Supplementary Fig. 45 The inherent temperature-dependent photoluminescence properties of RTP SiO_2 NPs
Supplementary Fig. 46 The temperature-dependent transmittance characteristics of the cured R-PC gel
Supplementary Fig. 47 The inherent temperature effects of FL and RTP of R-PC gel
Supplementary Fig. 48 Scattering enhancement behavior of poorly ordered PC gel
Supplementary Fig. 49 RTP thermochromic properties of R-PC gel
Supplementary Fig. 50 Scattering enhancement at low temperatures leads to PBG destruction
Supplementary Table 1 Summary of RTP lifetimes of the RTP SiO_2 NPs obtained at different temperatures (77-347 K)
Supplementary Table 2 Summary of RTP lifetimes of the RTP SiO_2 NPs obtained at different calcination temperatures
Supplementary Table 3 Summary of the PLQYs of RTP SiO_2 NPs obtained at different calcination temperatures
Supplementary Table 4 The mass and yield of RTP SiO_2 NPs prepared at different scales
Supplementary References

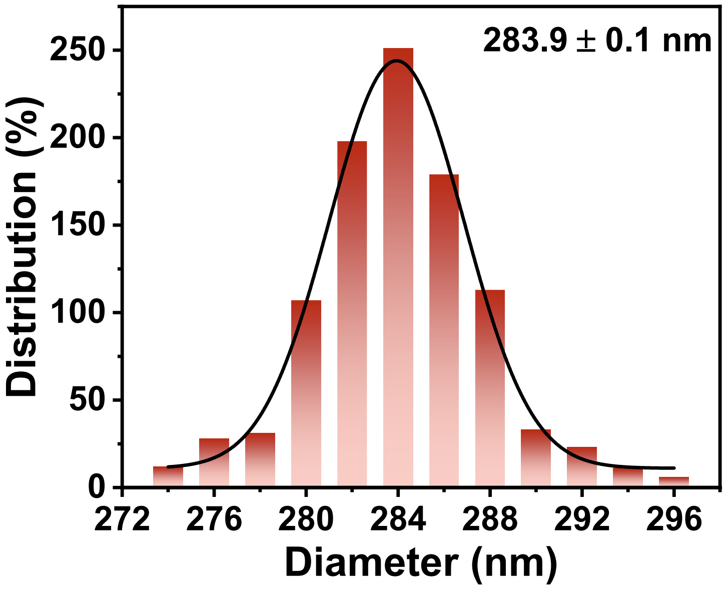

Supplementary Fig. 1: Diameter distribution of RTP SiO_2 NPs

Particle size distribution of RTP SiO_2 NPs measured by 1,000 particles in TEM. Source data are provided as a Source Data file.

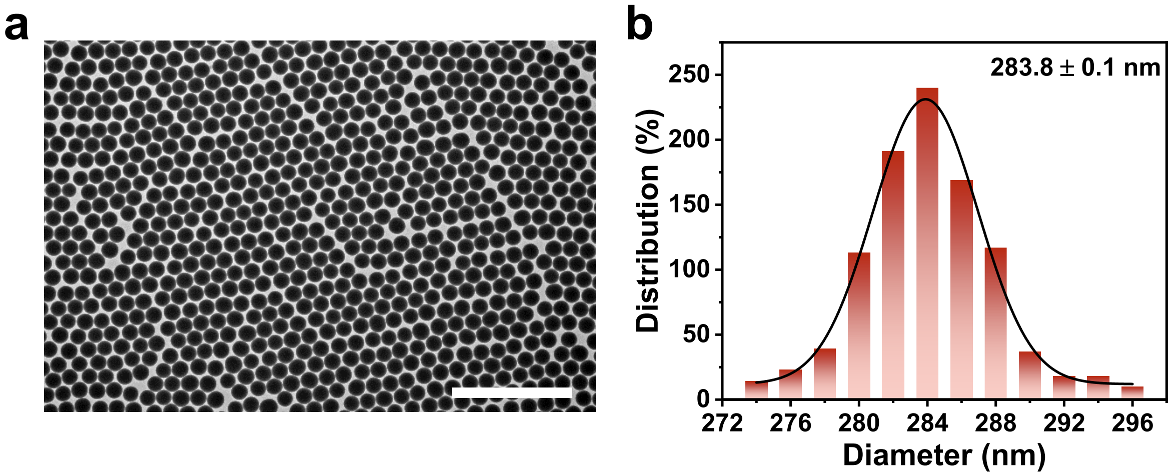

Supplementary Fig. 2: Morphology and diameter of uncalcined glucose doped SiO_2 NPs

a TEM image of uncalcined glucose molecules doped SiO_2 NPs (scale bar: 2 μm). **b** The particle size distribution of uncalcined glucose molecules doped SiO_2 NPs measured by 1,000 particles in TEM. Source data are provided as a Source Data file.

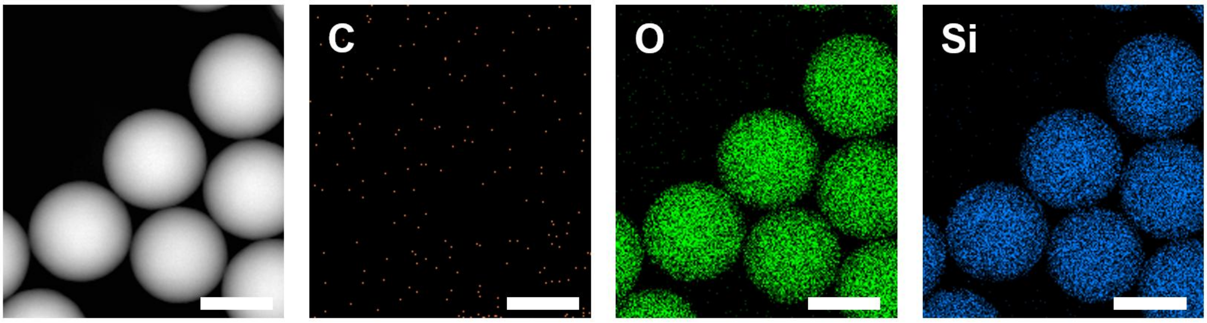

Supplementary Fig. 3: Distribution of chemical elements of pure SiO_2 NPs

HAADF-STEM and EDS elemental mapping images of pure SiO_2 NPs (scale bar: 200 nm).

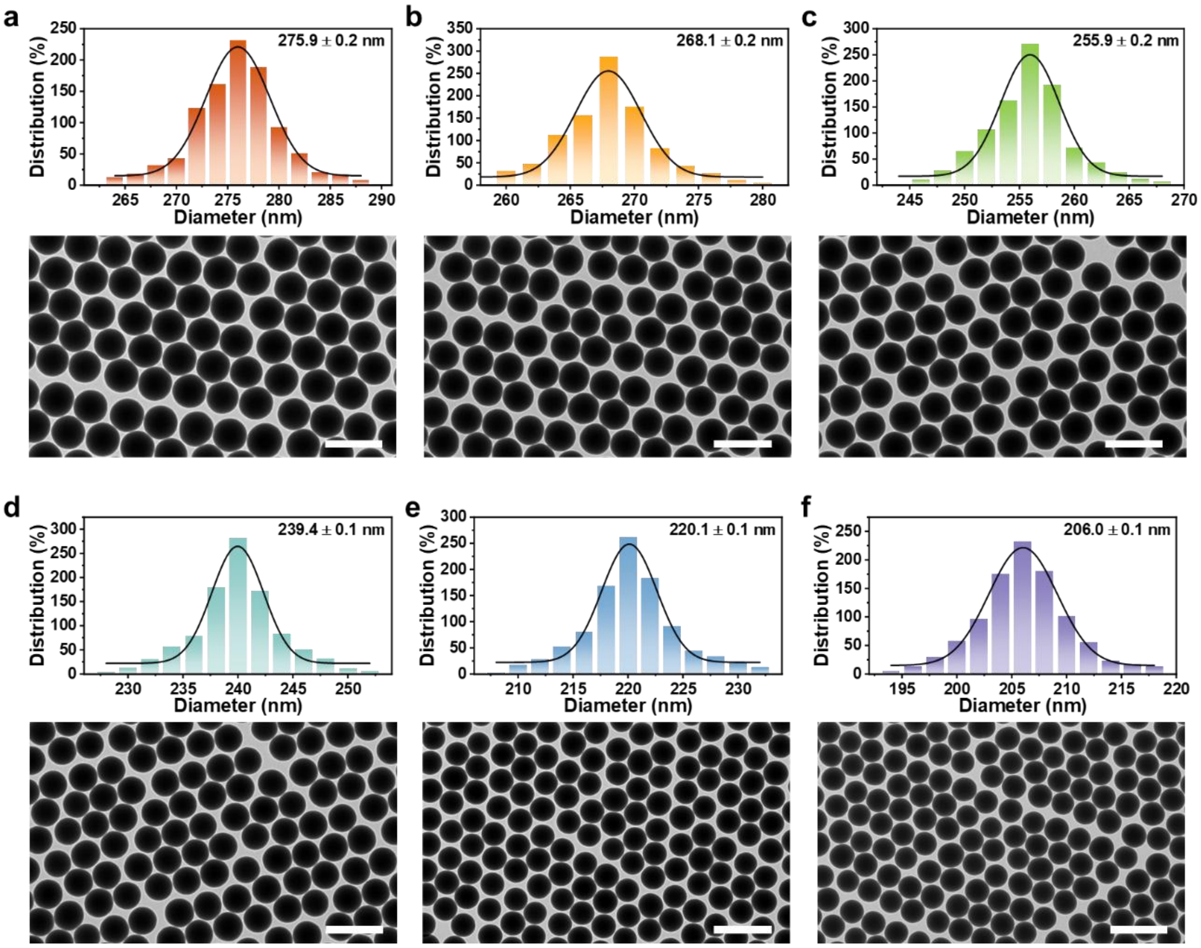

Supplementary Fig. 4: The morphology and diameter distribution of RTP SiO_2 NPs with different particle sizes

TEM images of synthesized RTP SiO_2 NPs with different particle sizes and corresponding particle size distribution measured by 1,000 particles, (scale bar: 500 nm). Source data are provided as a Source Data file.

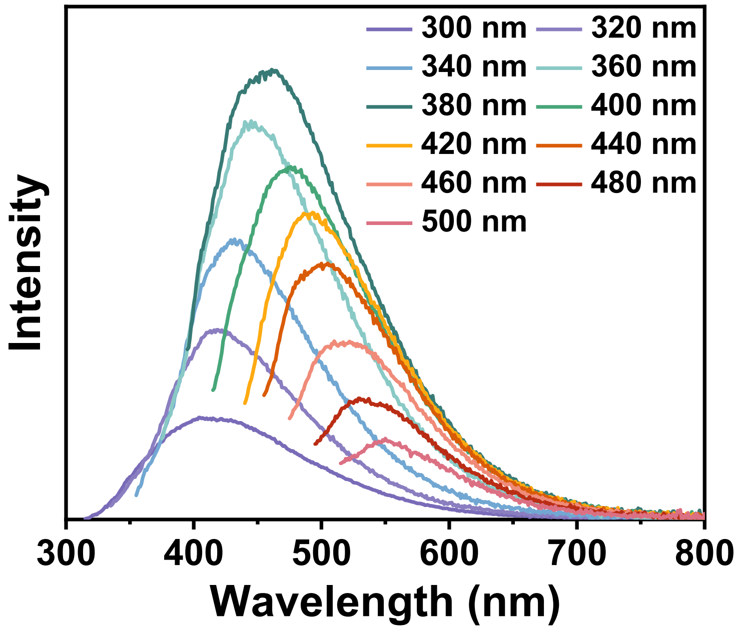

Supplementary Fig. 5: Fluorescence excitation dependence of RTP SiO_2 NPs

Excitation-dependent FL spectra of RTP SiO_2 NPs. Source data are provided as a Source Data file.

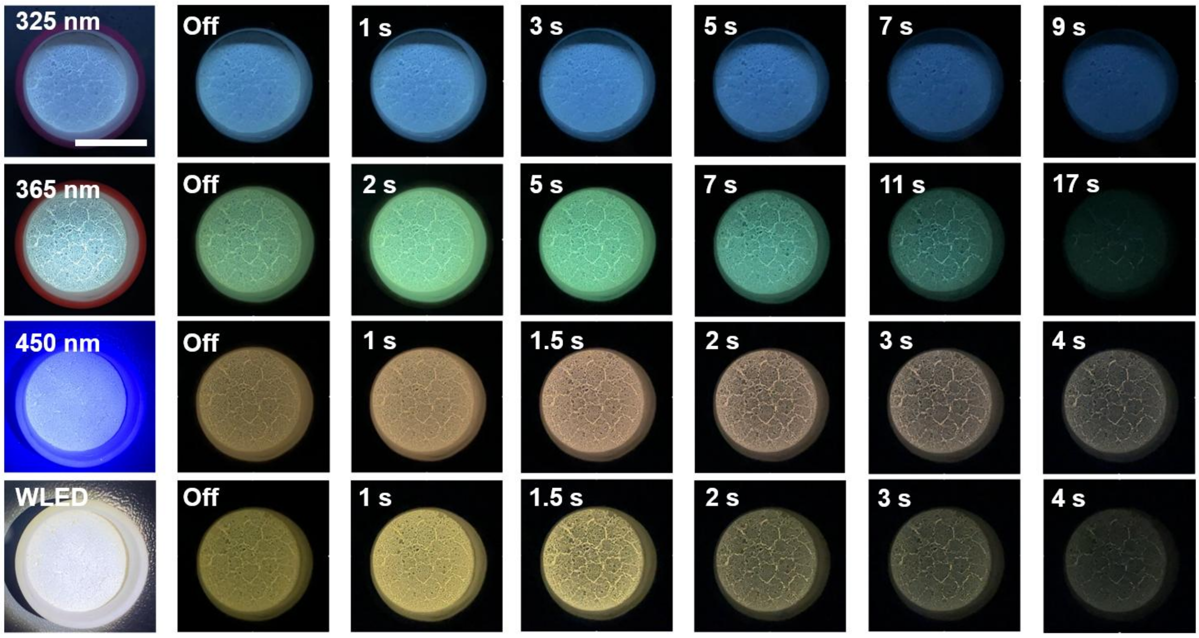

Supplementary Fig. 6: Afterglow photographs of RTP SiO_2 NPs powders

The afterglow photographs of RTP SiO_2 NPs taken at different time delay intervals and different excitation, scale bar: 5 cm.

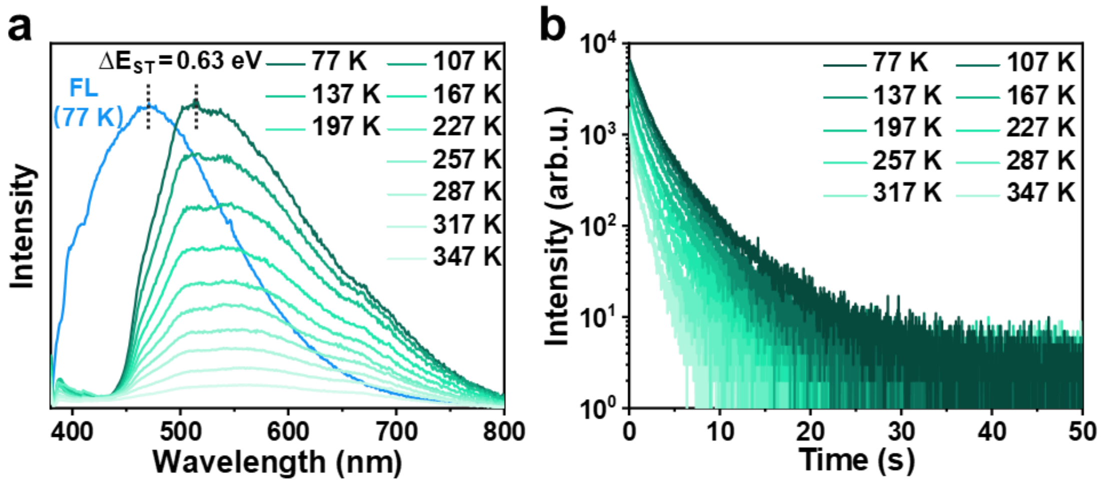

Supplementary Fig. 7: Temperature dependence of afterglow properties of RTP SiO_2 NPs

a Temperature-dependent afterglow spectra and **b** Temperature-dependent time-resolved afterglow decay spectra of RTP SiO_2 NPs under 365 nm excitation. Source data are provided as a Source Data file.

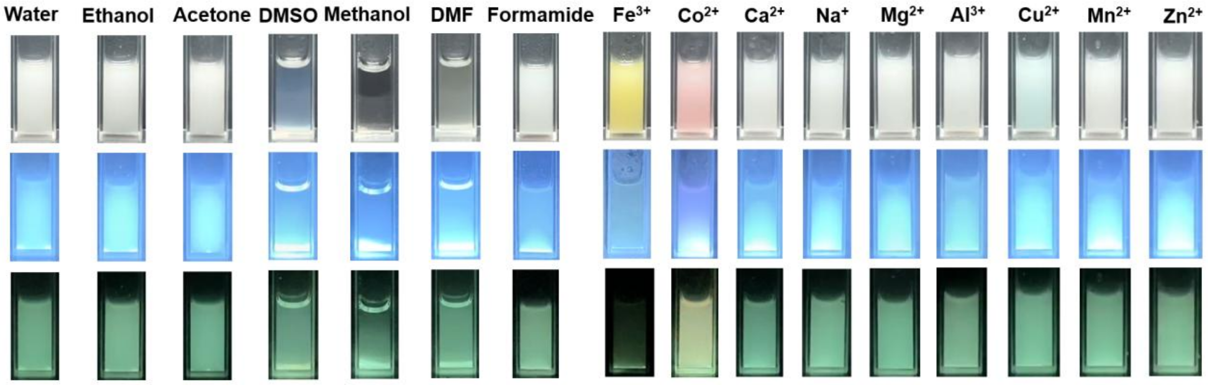

Supplementary Fig. 8: Photos of RTP SiO_2 NPs in various environments

The afterglow photographs of RTP SiO_2 NPs dispersed in different organic solvents and metal ion solutions (1 M).

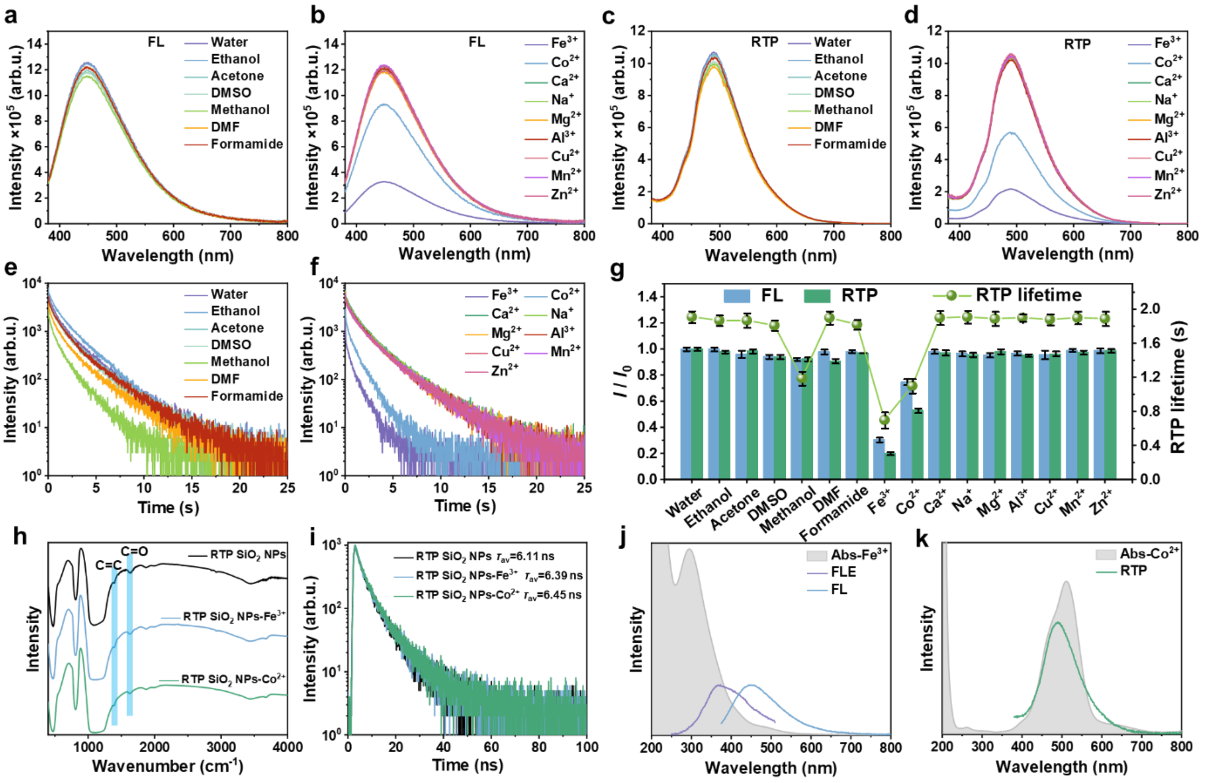

Supplementary Fig. 9: Systematic study on the optical stability and mechanism of RTP SiO_2 NPs

FL spectra of RTP SiO_2 NPs dispersed in **a** different solvents and **b** various metal ion solutions. RTP spectra of RTP SiO_2 NPs dispersed in **c** different solvents and **d** various metal ion solutions. RTP decay curve of RTP SiO_2 NPs dispersed in **e** different solvents and **f** various metal ion solutions. **g** The optical stability of the summarized RTP SiO_2 NPs in different environments, where the I_0 and I represent the emission intensity of RTP SiO_2 NPs dispersed in water and in different environments, respectively, the error bars represent the standard deviation derived from five independent experimental measurements. **h** FT-IR spectra of RTP SiO_2 NPs water dispersion in the presence and absence of Fe^{3+} and Co^{2+}. **i** FL decay profile of RTP SiO_2 NPs water dispersion in the presence and absence of Fe^{3+} and Co^{2+}. **j** The UV-vis absorption spectra of Fe^{3+}, and the FLE and FL spectra of the RTP SiO_2 NPs water dispersion. **k** The UV-vis absorption spectra of Co^{2+}, and RTP spectra of the RTP SiO_2 NPs water dispersion. Source data are provided as a Source Data file.

Discussion: Systematic exploration of the optical stability of RTP SiO_2 NPs in the presence of different organic solvents and different kinds of metal ionic interferences with the concentration of 1 M under identical conditions. Supplementary Fig. 9a-d show the Fe^{3+} and Co^{2+} can induce FL and RTP quenching for RTP SiO_2 NPs. Meanwhile, Fe^{3+} and Co^{2+} also have a significant impact on the RTP lifetime of RTP SiO_2 NPs (Supplementary Fig. 9e and f). It is worth mentioning that RTP SiO_2 NPs also exhibit a short RTP lifetime in methanol, which is due to low solubility of RTP SiO_2 NPs in methanol (Supplementary Fig. 9g). Generally, the quenching of fluorophores by metal ions mainly involves static quenching, dynamic quenching and inner filter effect.[1] Firstly, the FT-IR spectra of RTP SiO_2 NPs in the presence and absence of Fe^{3+} and Co^{2+} show that no obvious shift and no new vibration absorption sites appear (Supplementary Fig. 9h), indicating that no functional groups are formed or adhered to the surface of RTP SiO_2 NPs, thus excluding static quenching.[2] To seek the reason for the quenching phenomenon, the fluorescence lifetime of RTP SiO_2 NPs with and without Fe^{3+} and Co^{2+} are measured. As shown in Supplementary Fig. 9i, the fluorescence lifetime of RTP SiO_2 NPs in the absence and presence Fe^{3+} and Co^{2+} are almost unchanged. The inner filter effect typically requires that the absorption band of the quencher overlap with the excitation or emission band of the fluorophore, resulting in a bursting behavior by absorbing the excitation or emission light of the fluorophore.[3] As shown in the Supplementary Fig. 9j and k, the absorption spectra of Fe^{3+} and Co^{2+} ions overlap with the excitation and RTP emission peaks of RTP SiO_2 NPs, respectively, indicating a typical inner filter effect.

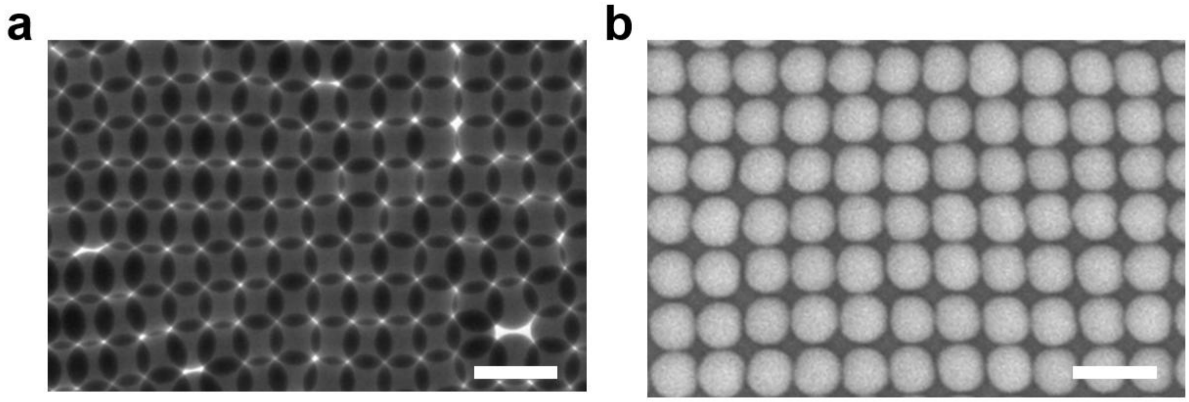

Supplementary Fig. 10: Morphological characterization of RTP SiO_2 NPs after self-assembly

a TEM and **b** SEM images of (100) plane of RTP SiO_2 NPs stacked in fcc structure. (scale bar: 500 nm).

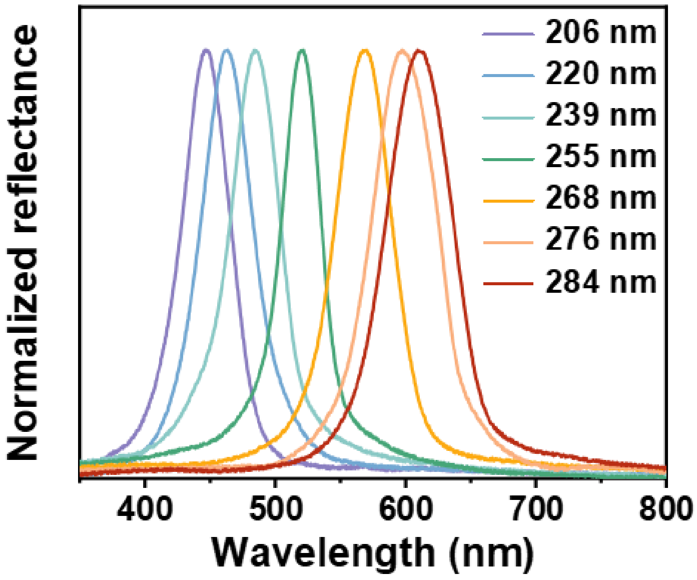

Supplementary Fig. 11: Optical characterization of PCs constructed from RTP SiO_2 NPs with different particle sizes

The normalized reflection spectra of the multimodal PCs assembled by RTP SiO_2 NPs with different diameters, θ=90°. Source data are provided as a Source Data file.

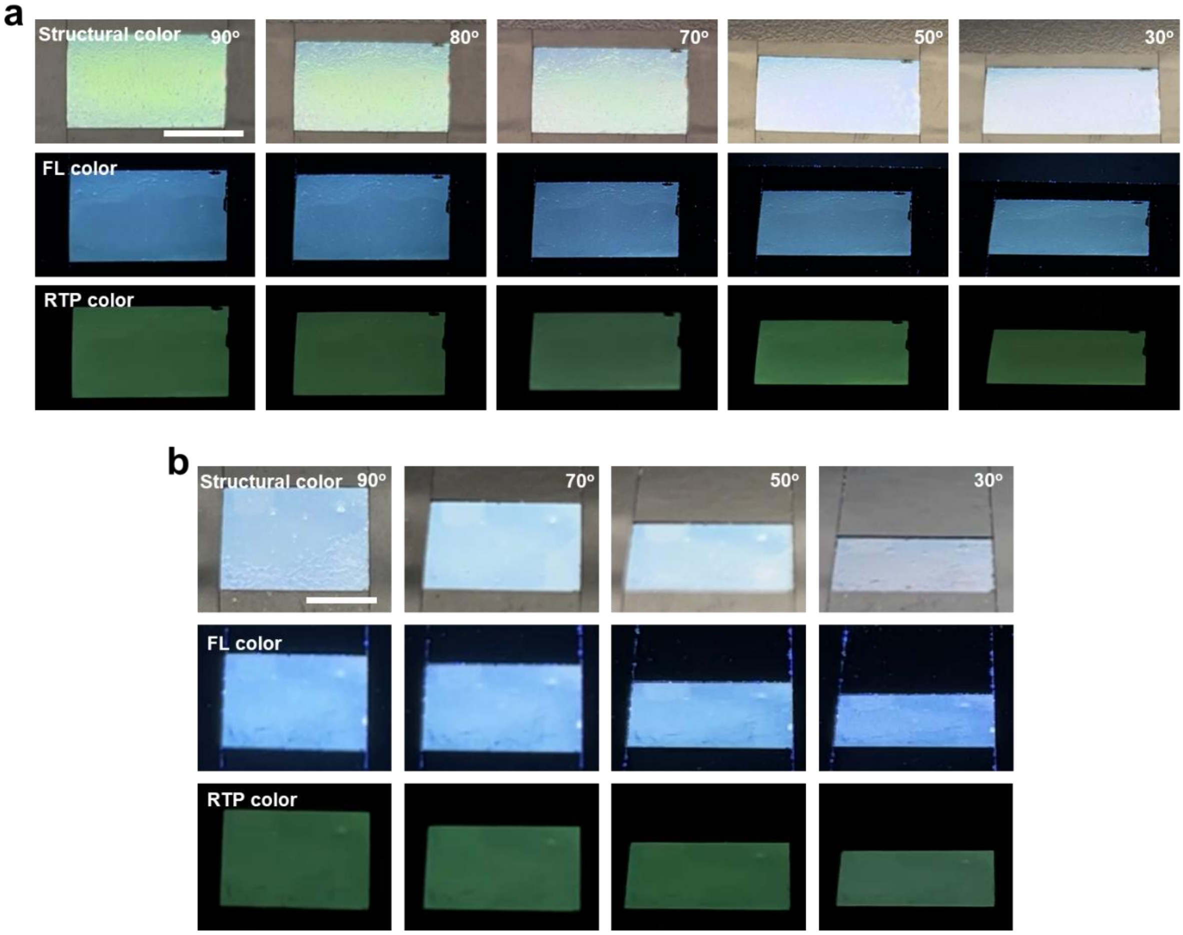

Supplementary Fig. 12: Photographs of three optical morphologies of multimode PCs

The photographs of the angle-dependent structural colors, FL and RTP of the multimodal PCs self-assembled by NPs diameter of **a** 239 nm and **b** 220 nm under daylight, 365 nm UV lamp on and off, respectively, scale bar: 1 cm.

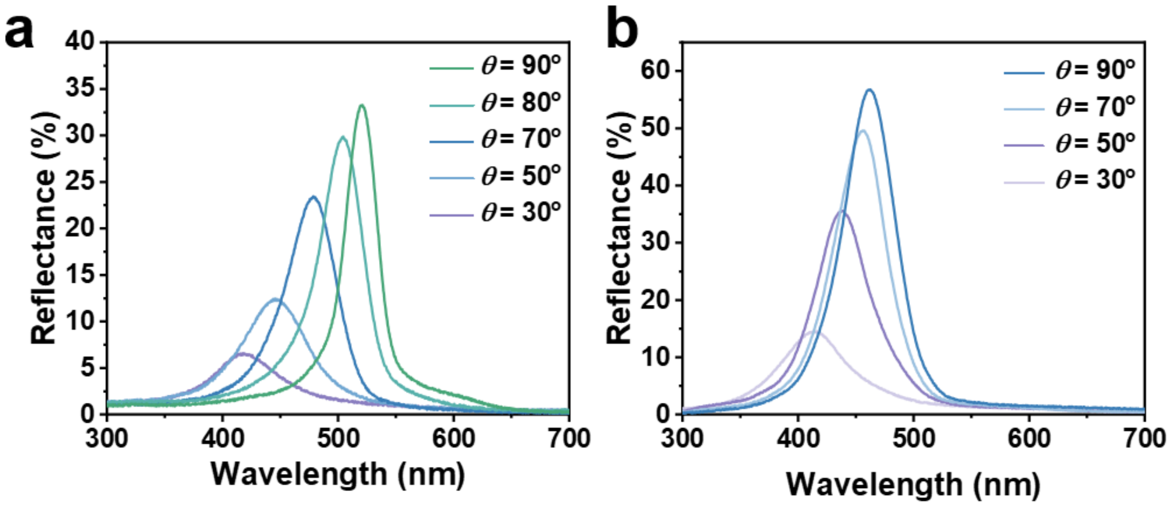

Supplementary Fig. 13: Angle-dependent reflection spectra of multimode PCs

Angle-dependent reflection spectra of the multimodal PCs self-assembled by RTP SiO_2 NPs with diameter of **a** 239 nm and **b** 220 nm. Source data are provided as a Source Data file.

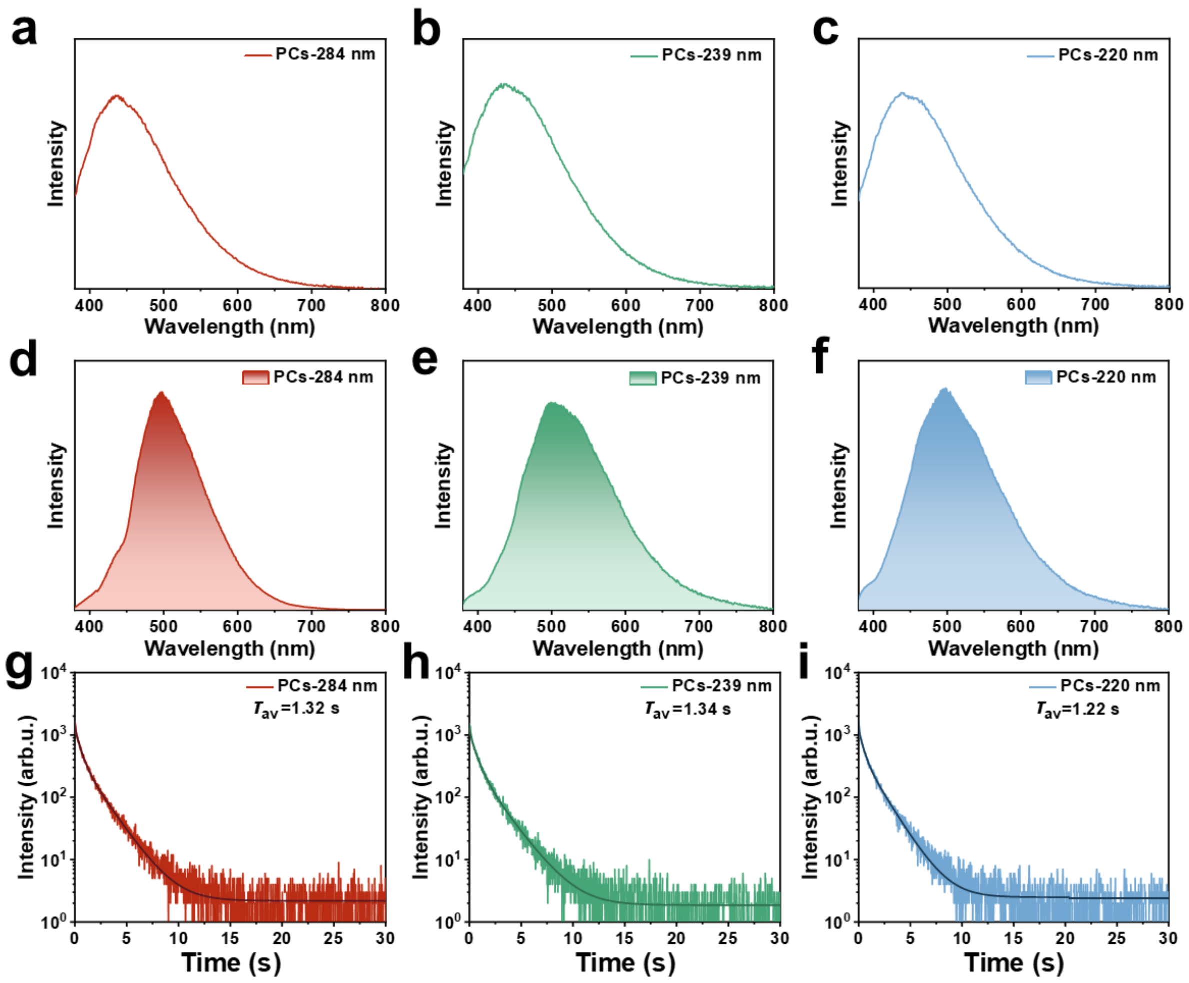

Supplementary Fig. 14: FL and RTP characteristics of multimode PCs assembled with different particle sizes of RTP SiO_2 NPs

a-c The FL spectra of the multimodal PCs self-assembled by RTP SiO_2 NPs with different diameter. **d-f** The RTP spectra of the multimodal PCs self-assembled by RTP SiO_2 NPs with different diameter. **g-h** The RTP lifetime decay profile of the multimodal PCs self-assembled by RTP SiO_2 NPs with different diameter. Source data are provided as a Source Data file.

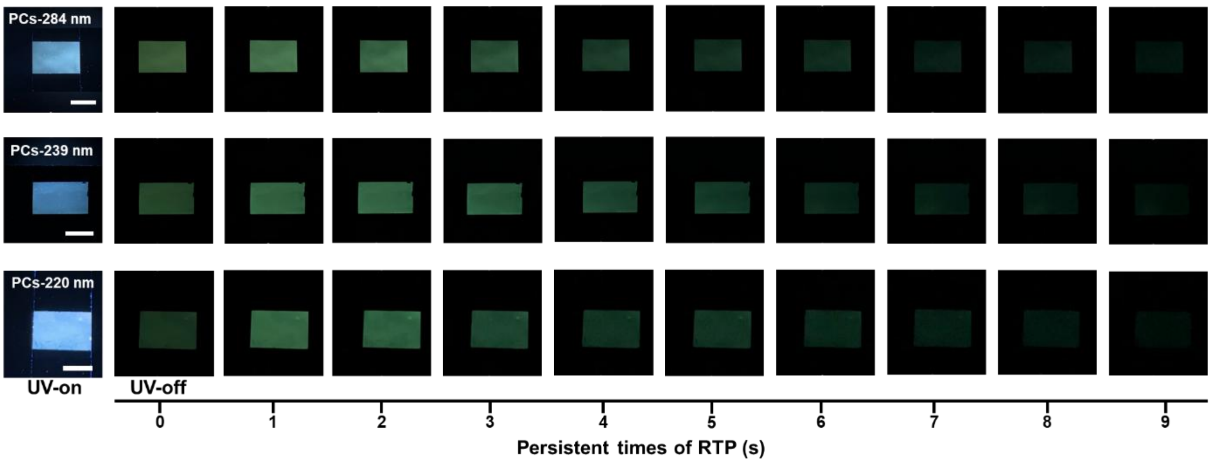

Supplementary Fig. 15: Afterglow photos of multimode PCs

The afterglow photographs of multimodal PCs self-assembled by RTP SiO_2 NPs with different diameter taken at different time delay intervals, scale bar: 1 cm.

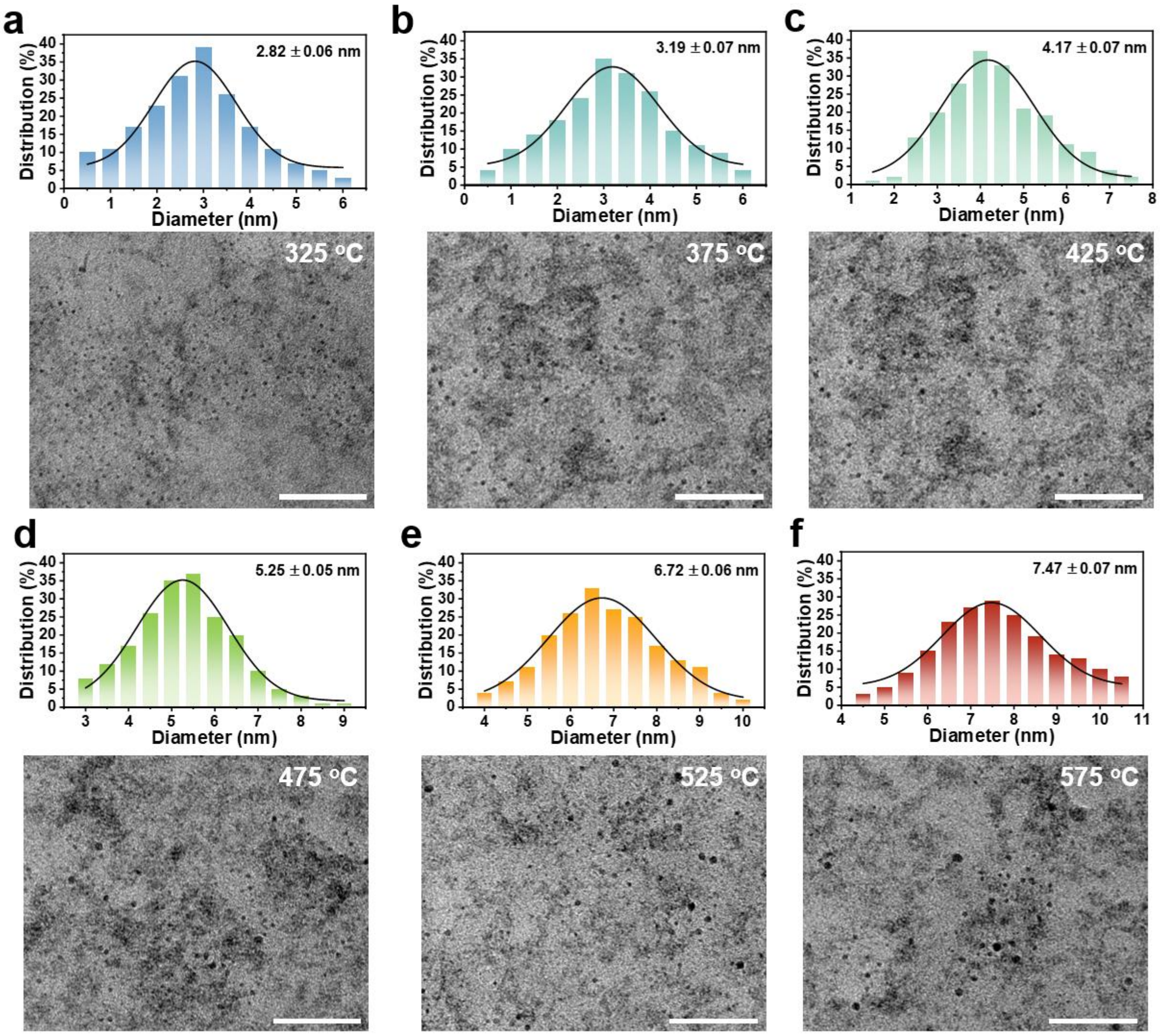

Supplementary Fig. 16: Morphology and particle size distribution of CDs generated in situ inside SiO_2 NPs

The TEM images of the RTP SiO_2 NPs generated at different calcination temperatures after hydrofluoric acid etching, as well as the histograms of the particle size distribution of CDs summarized from the TEM measured by 200 particles, (scale bar: 50 nm). Source data are provided as a Source Data file.

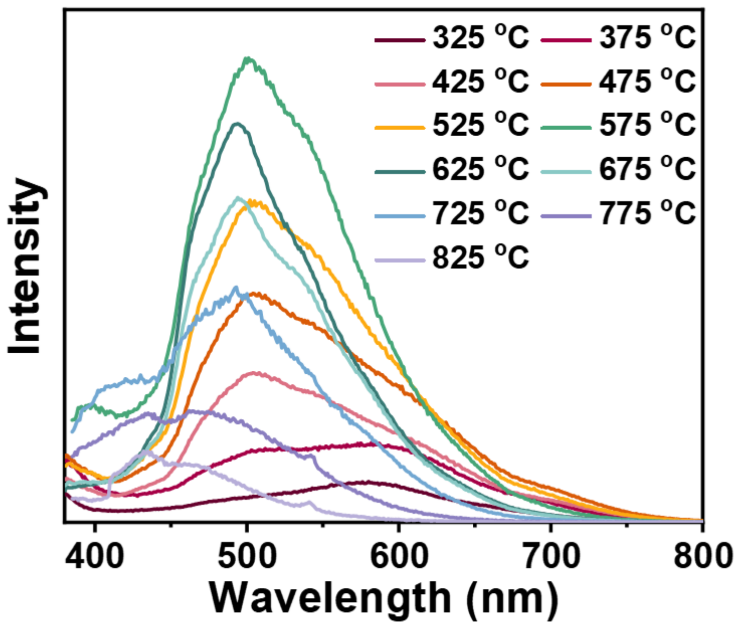

Supplementary Fig. 17: Calcination temperature dependent RTP characteristics of RTP SiO_2 NPs

The RTP spectra of RTP SiO_2 NPs obtained at different calcination temperatures. Source data are provided as a Source Data file.

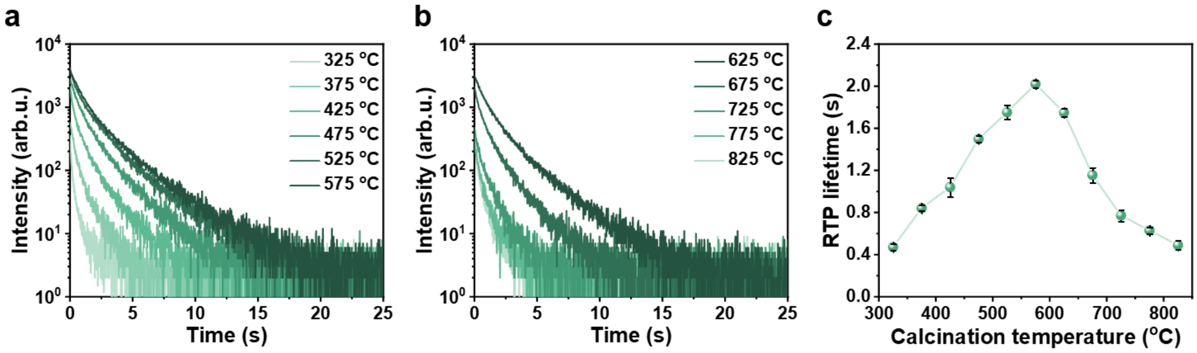

Supplementary Fig. 18: Relationship between RTP lifetime of RTP SiO_2 NPs and calcination temperature

a and **b** The RTP lifetime decay spectra of RTP SiO_2 NPs obtained at different calcination temperatures (diameter: 284 nm). **c** The relationship between RTP lifetime and calcination temperature, the error bars represent the 95% confidence interval calculated from five independent experimental measurements. Source data are provided as a Source Data file.

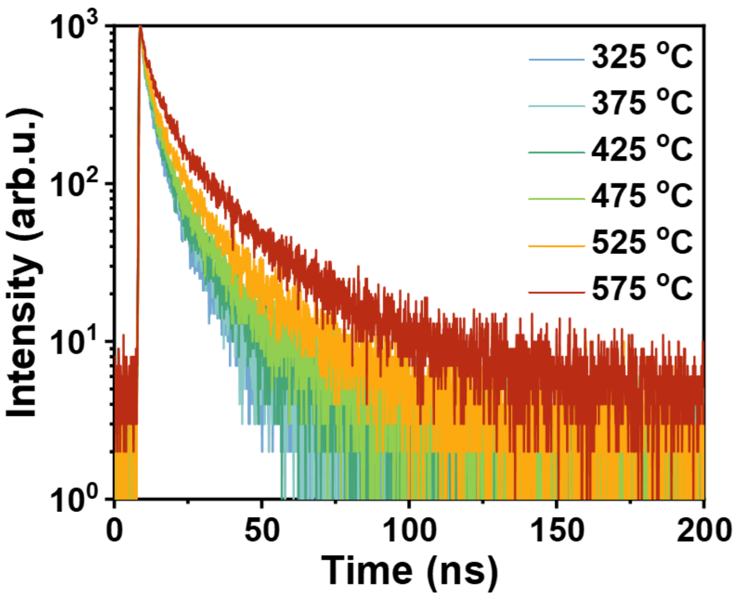

Supplementary Fig. 19: Relationship between FL lifetime of RTP SiO_2 NPs and calcination temperature

The FL lifetime decay spectra of RTP SiO_2 NPs obtained at different calcination temperatures (diameter: 284 nm). Source data are provided as a Source Data file.

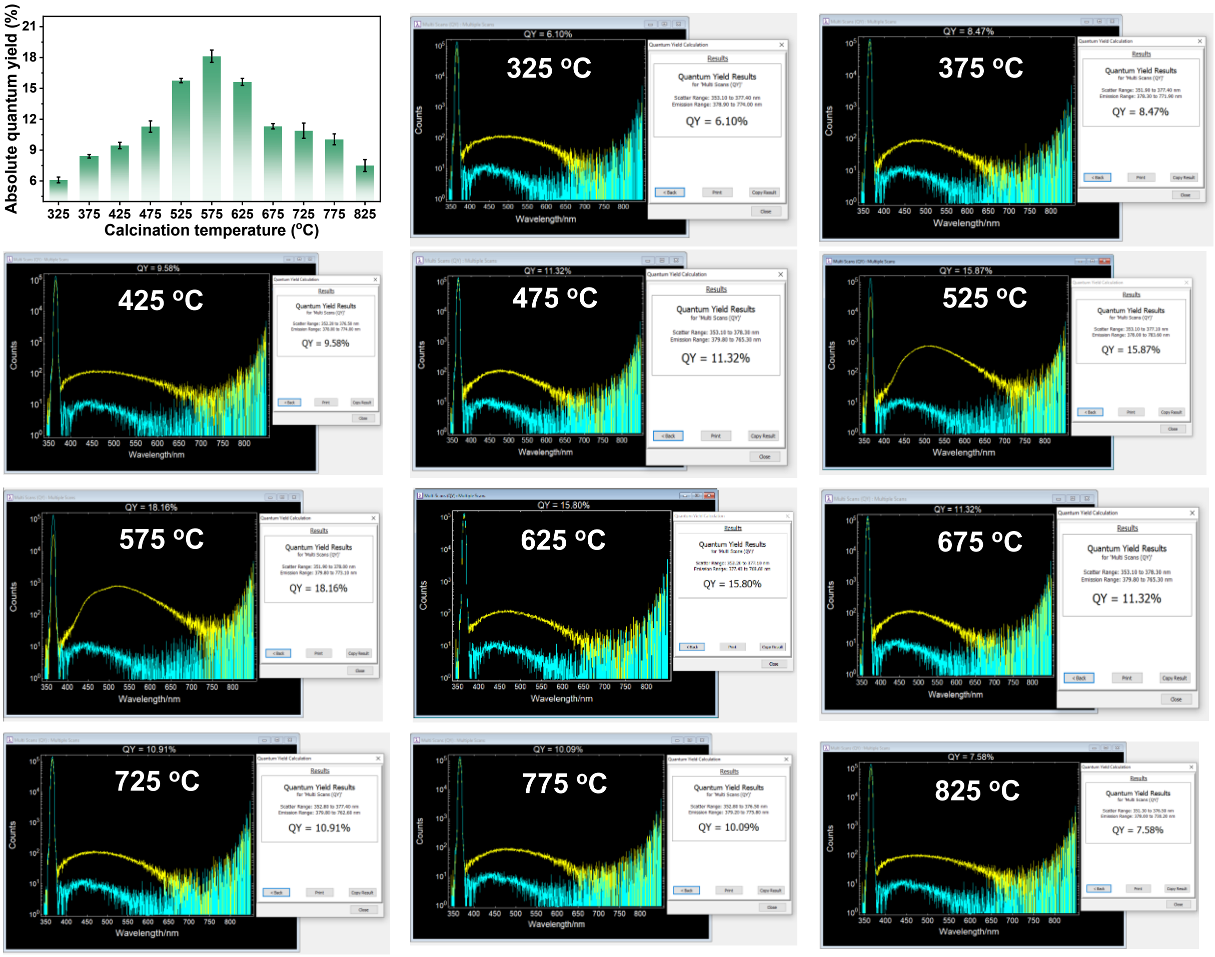

Supplementary Fig. 20: PLQY of RTP SiO_2 NPs

The relationship between the absolute quantum yield (PLQY) and calcination temperatures, the error bars represent the 95% confidence interval calculated from five independent experimental measurements. Source data are provided as a Source Data file.

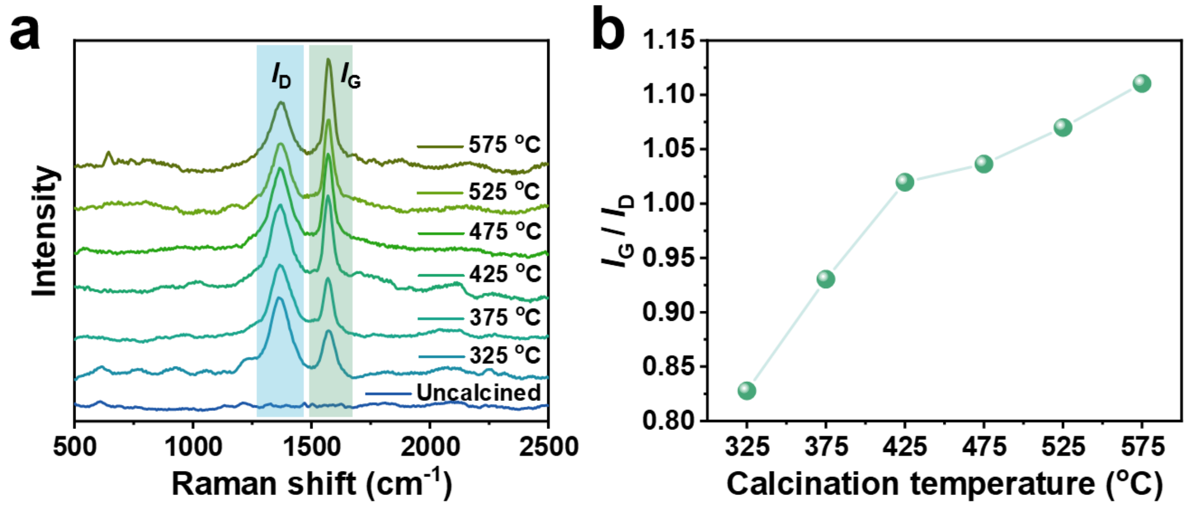

Supplementary Fig. 21: The degree of crystallization of in situ CDs in RTP SiO_2 NPs

a The Raman spectra of RTP SiO_2 NPs obtained at different calcination temperatures. **b** The relationship between I_G/I_D and calcination temperatures. Source data are provided as a Source Data file.

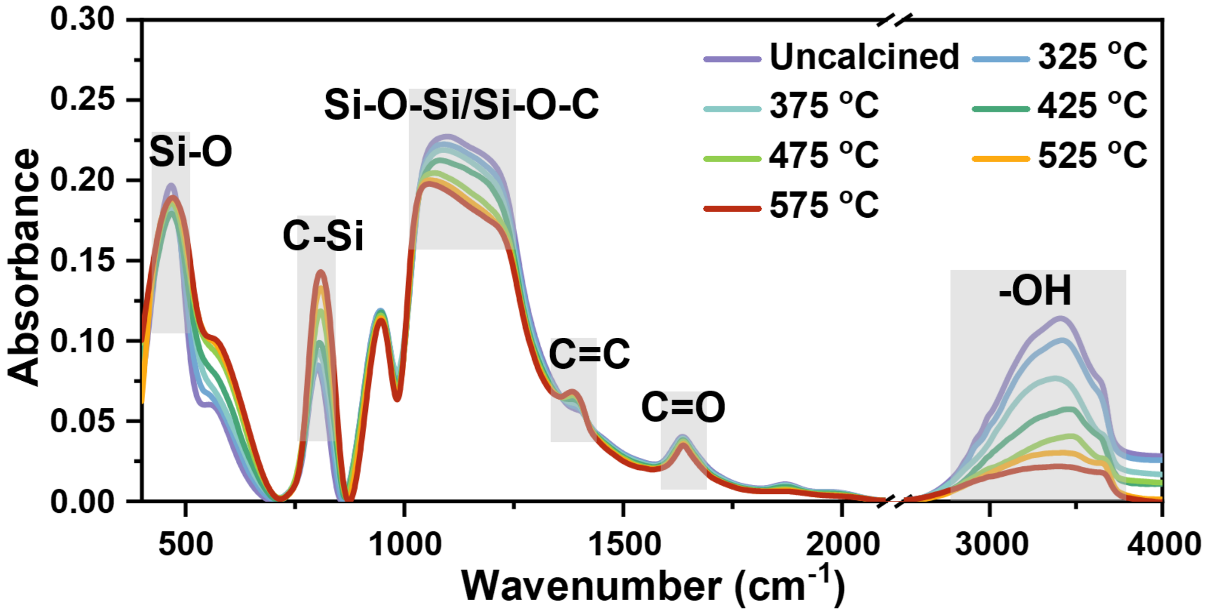

Supplementary Fig. 22: FT-IR spectra of RTP SiO_2 NPs

The FT-IR spectra of RTP SiO_2 NPs obtained at different calcination temperatures. Source data are provided as a Source Data file.

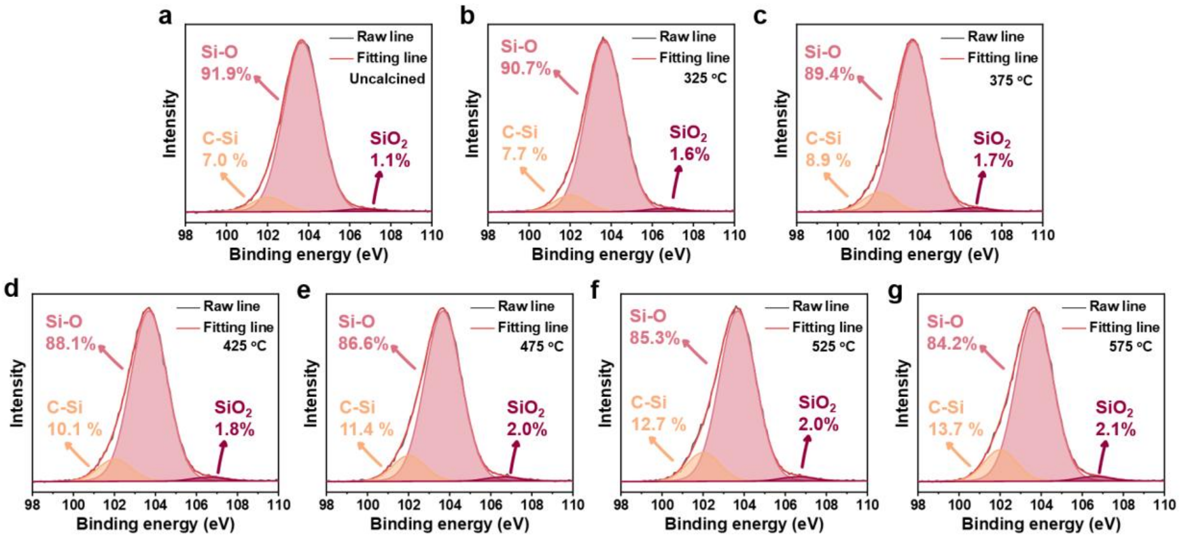

Supplementary Fig. 23: High-resolution Si 2*p* results of RTP SiO_2 NPs

The high-resolution Si 2*p* results of RTP SiO_2 NPs obtained at different calcination temperatures. Source data are provided as a Source Data file.

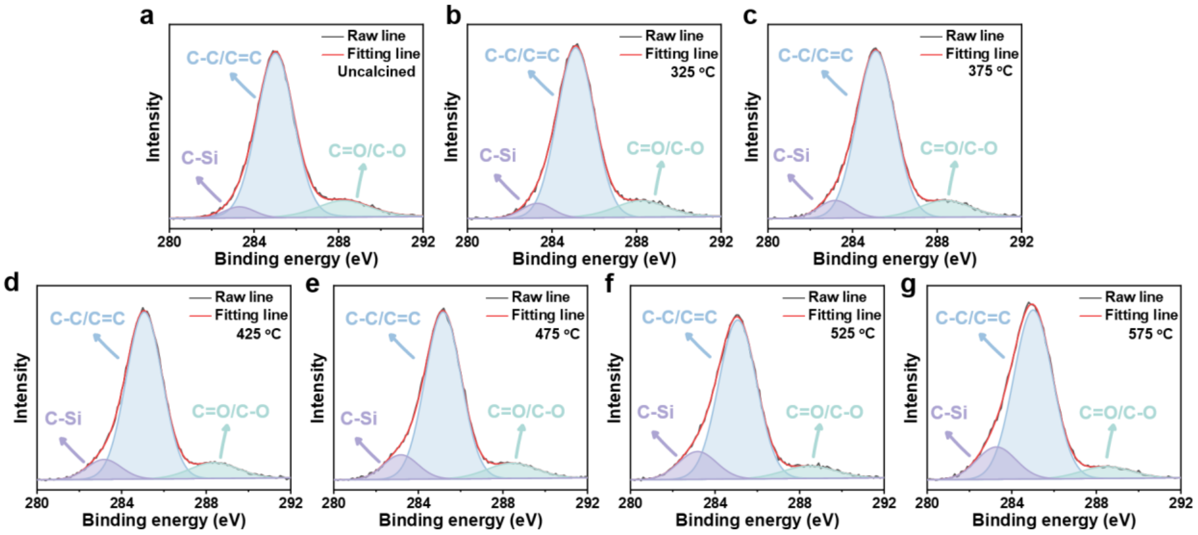

Supplementary Fig. 24: High-resolution C 1*s* results of RTP SiO_2 NPs

The high-resolution C 1*s* results of RTP SiO_2 NPs obtained at different calcination temperatures. Source data are provided as a Source Data file.

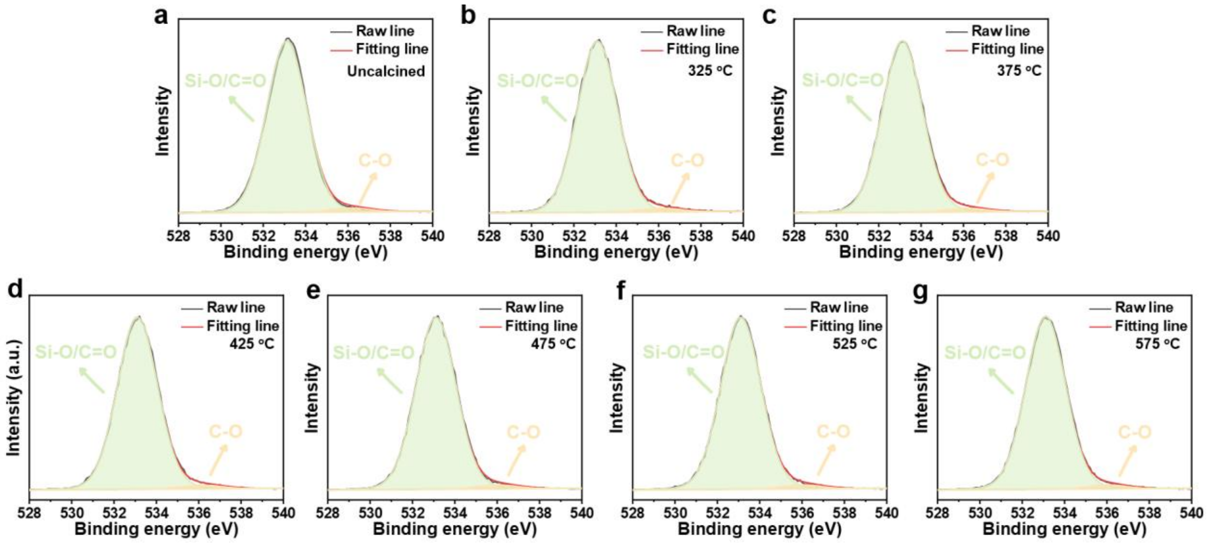

Supplementary Fig. 25: High-resolution O 1*s* results of RTP SiO_2 NPs

The high-resolution O 1*s* results of RTP SiO_2 NPs obtained at different calcination temperatures.

Source data are provided as a Source Data file.

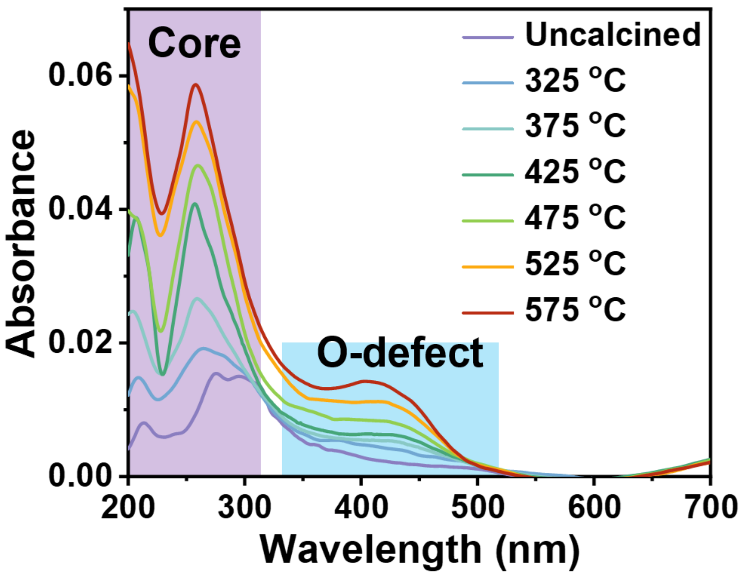

Supplementary Fig. 26: Absorption characteristic of RTP SiO_2 NPs

UV-vis absorption spectra of RTP SiO_2 NPs obtained at different calcination temperatures. Source data are provided as a Source Data file.

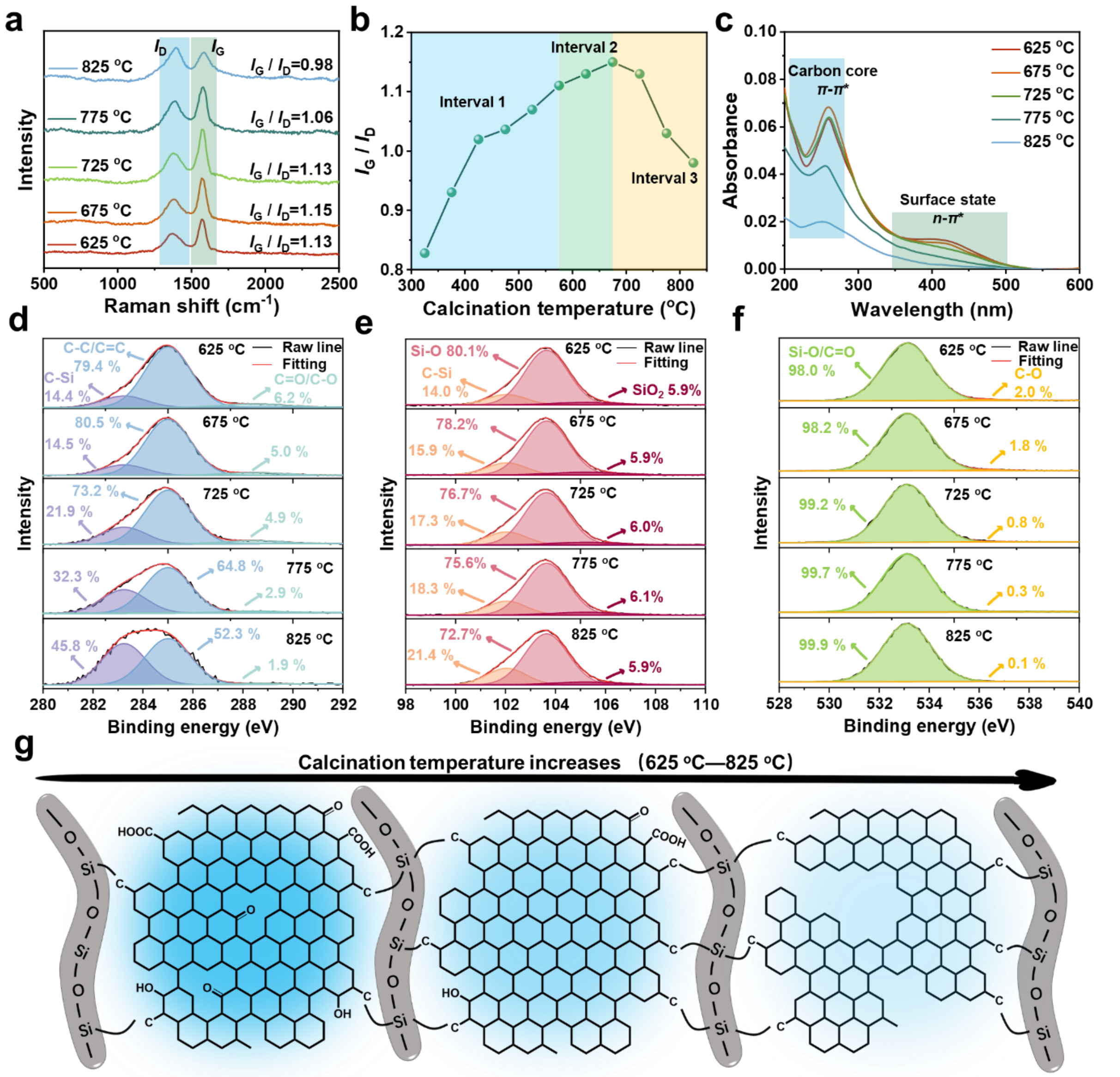

Supplementary Fig. 27: Exploration of the mechanism of decreased luminescence efficiency of RTP SiO_2 NPs calcined at excessively high temperatures

a The Raman spectra of RTP SiO_2 NPs obtained at different calcination temperatures. **b** The relationship between I_G/I_D and calcination temperatures. **c** UV-vis absorption spectra of RTP SiO_2 NPs obtained at different calcination temperatures. The high-resolution **d** Si 2*p*, **e** C 1*s* and **f** O 1*s* results of RTP SiO_2 NPs obtained at different calcination temperatures. **g** A plausible mechanism for the PLQY decrease in RTP SiO_2 NPs obtained under excessive temperature calcination. Source data are provided as a Source Data file.

Discussion: In order to explore the reduction of PLQY and optical quality caused by excessive high-temperature calcination, we conducted a systematic exploration. As the calcination temperature increased from 625 to 825 ºC, the intensity ratio (I_G/I_D) of crystalline G band and disordered D band in Raman spectra shows a trend of increasing first and then decreasing (Supplementary Fig. 27a). Combining the Raman spectra measured in the range of 325-575 ºC for summary, we found that I_G/I_D shows a non-monotonic changing trend, gradually increasing in the range of 325-675 ºC, reaching the peak at 675 ºC, and then significantly decreasing in the range of 675-825 ºC (Supplementary Fig. 27b). Therefore, we speculate that the influence of calcination temperature on the in-situ formation of CDs inside SiO_2 can be divided into three intervals: in the first stage, as the temperature increases from 325 to 575 ºC, the organic small molecules SiO_2 undergo carbonization and crystallization to form CDs, which has the optimal optical properties at the calcination temperature of 575 ºC. The CDs generated at 575 ºC has a high crystallinity and a rich variety of chemical defects (O-related defects) on the surface, which the defect states can enhance the ability to capture electrons and provide additional radiative recombination transition channels for electrons, improving the overall luminescence efficiency. As the temperature further increases (from 575 to 675 ºC), the crystallinity of CDs will further increase, but the chemical defects are damaged due to the high temperature, thus the luminous efficiency begins to decrease. However, when the temperature further increases and exceeds 675 ºC, the structure of CDs is cracked by high temperature, resulting in a rapid decline in optical performance.

To verify this hypothesis, we have measured the UV-Vis absorption spectra and XPS spectra of RTP SiO_2 NPs generated at calcination temperatures ranging from 625 to 825 ºC. As shown in Supplementary Fig. 27c, as the calcination temperature increased, the broad absorption band in the range of approximately 350-500 nm, attributed to the n-π* electronic transitions of surface defect states in the CDs, gradually diminished. This indicates that increased temperatures led to a reduction in the chemical defect states on the surfaces of CDs.

Additionally, for silica calcined at 775 and 825 ºC, the high-energy π-π* electronic transition absorption peak at ~258 nm, originating from the carbon cores of the CDs, is significantly weakened, suggesting structural degradation of the carbon cores. Furthermore, the fitted high-resolution C 1*s* results of RTP SiO_2 NPs generated at calcination temperatures shown in Supplementary Fig. 27d demonstrate that they both contain three peaks at about 282.2, 285.0 and 288.5 eV, which are assigned to C-Si, C-C/C=C and C=O/C-O, respectively. The fitting results show an increase in the relative content of C-Si bonds and a decrease in C-C/C=C and C=O/C-O. Although the content of C-Si continues to increase, the excessively high calcination temperature will lead to the destruction of the chemical defect state, and cause the partial cleavage of the CDs, which ultimately leading to a decrease in the overall optical performance. The corresponding high-resolution Si 2*p* and O 1*s* fitting results further confirm this view (Supplementary Figs. 27e-f). In conclusion, based on the above discussion, when the calcination temperature exceeds 625 ºC, the decrease in quantum yield of RTP SiO_2 NPs is mainly due to the reduction of chemical defect states of internal CDs and the decomposition of their own structure (Supplementary Fig. 27g).

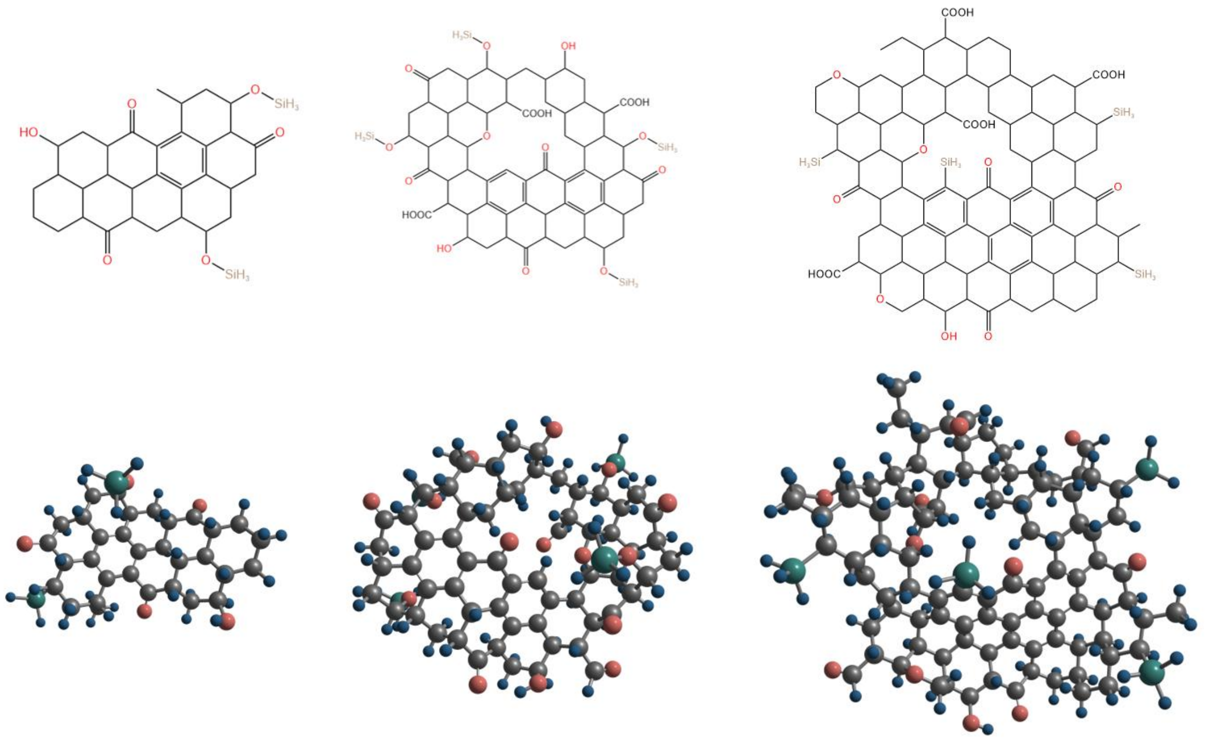

CDs-325 ºC **CDs-475 ºC** **CDs-575 ºC**

Supplementary Fig. 28: Three CDs calculation models based on HR-TEM and chemical characterization

Calculation model for CDs generation under calcination at 325 ºC, 475 ºC and 575 ºC based on HR-TEM and FT-IR results, where black-gray represents carbon atoms, orange-red represents oxygen atoms, dark green represents silicon atoms, and cyan-blue represents hydrogen atoms.

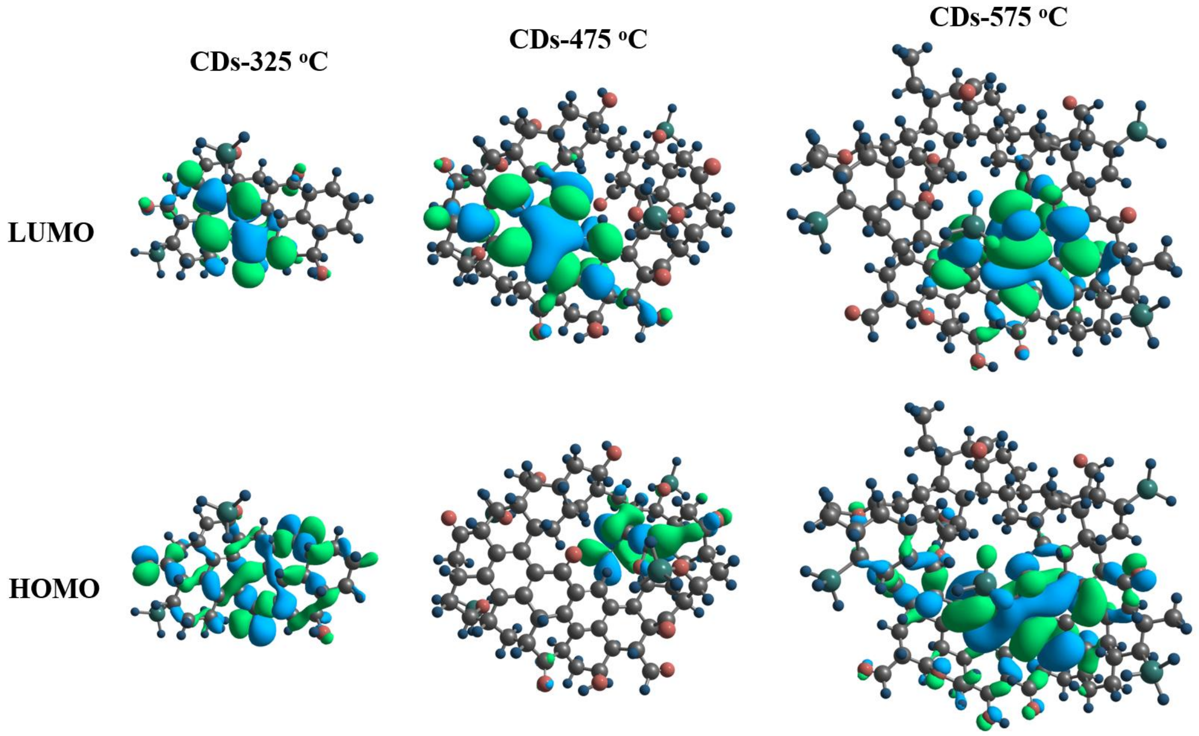

Supplementary Fig. 29: Quantum chemical calculations for three kinds of CDs models

Quantum chemical calculations of HOMO and LUMO distributions for the models of CDs-325 °C, CDs-475 °C and CDs-575 °C. The light green and light blue balloons represent bonding and anti-bonding orbitals in the lowest unoccupied molecular orbital, respectively.

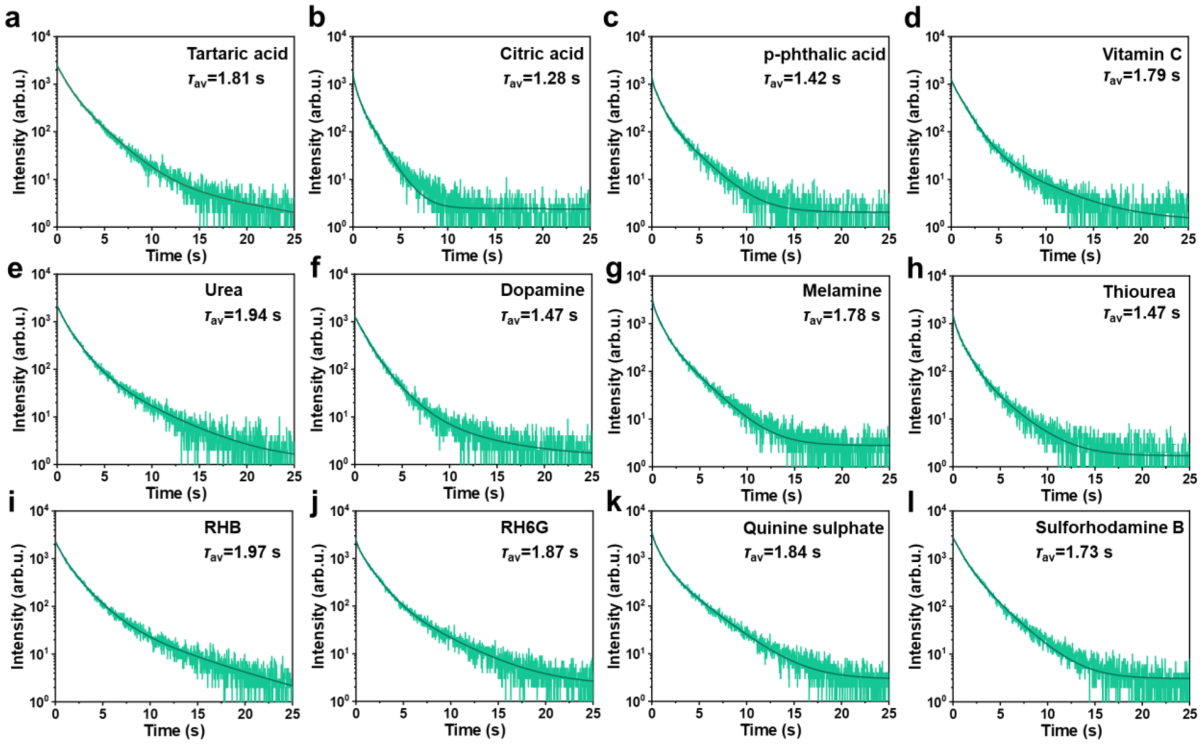

Supplementary Fig. 30: Results of the validation of the universality of the method

RTP lifetime decay spectra of RTP SiO_2 NPs with diverse doped types of organic small molecule. Source data are provided as a Source Data file.

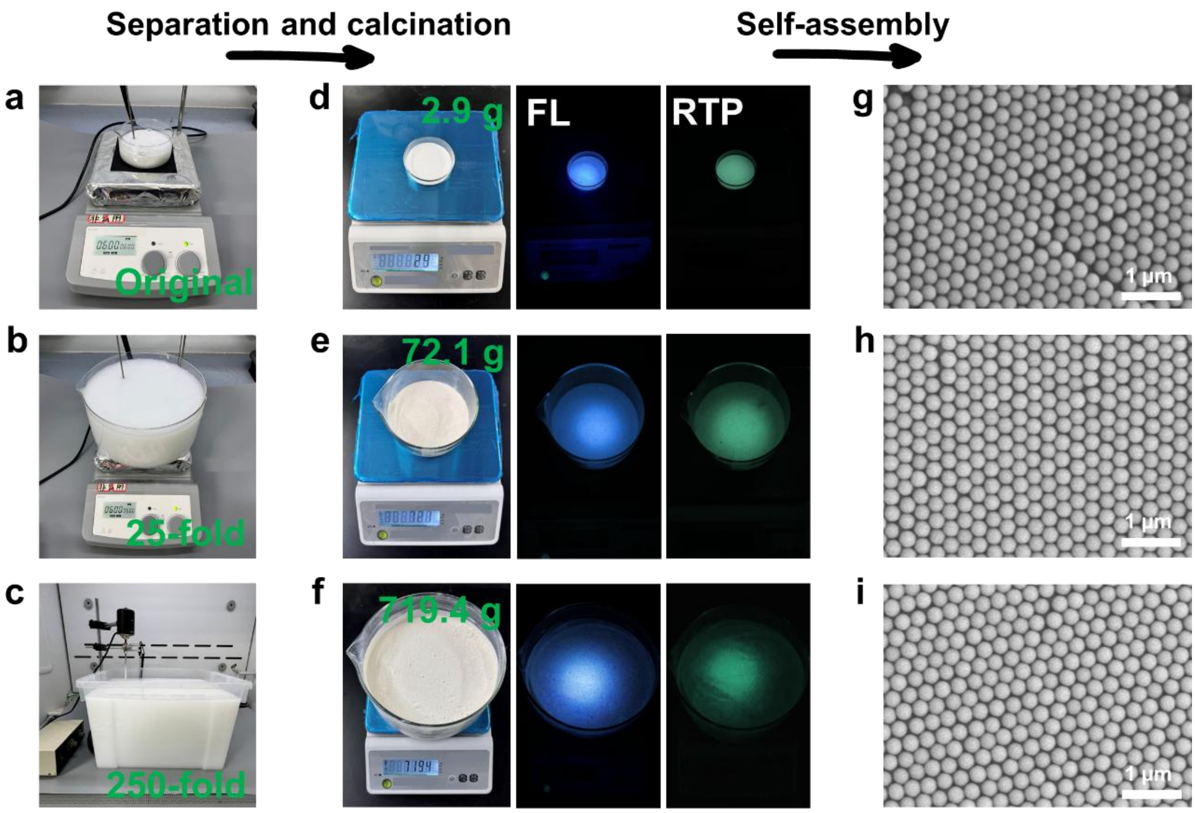

Supplementary Fig. 31: Demonstration of large-scale preparation of RTP SiO_2 NPs

a-c Photograph of RTP SiO_2 NPs prepared at magnifications of 1-fold, 25-fol and 250-fold. **d-f** The corresponding photographs of large-scale prepared RTP SiO_2 NPs under sunlight, UV radiation and after UV shut-off. **g-i** SEM images of RTP SiO_2 NPs prepared at different scales after self-assembly.

Discussion: In order to more intuitively and comprehensively demonstrate the capabilities of designed strategy for the large-scale preparation of self-assembled RTP SiO_2 NPs with a low dispersity in size, we conducted 25-fold and 250-fold proportional amplification experiments on the basis of the original preparation experiments (1-fold reference amount) for verification. As presented in Supplementary Fig. 31, through the two preparation steps of Stöber method and high-temperature calcination, the RTP SiO_2 NPs obtained at three different preparation scales all exhibit FL and RTP. Importantly, their morphology and self-assembly capability remain unaffected by the scaling of reaction precursors, demonstrating excellent scalability of our designed synthesis strategy. Notably, thanks to the high flexibility of the Stöber method for

production equipment, we have achieved laboratory-scale preparation at the hundred-gram level (700 g per batch). Furthermore, we use the product mass obtained from the original synthetic method (1-fold) as the criterion to evaluate the yield of large-scale preparation. Supplementary Table 4 demonstrates that even when scaling up the reaction system by 250-fold, the yield remains consistently above 99.2% with a batch-to-batch variation of less than 0.8%. These results strongly validate the exceptional scalability and process stability of this synthetic methodology.

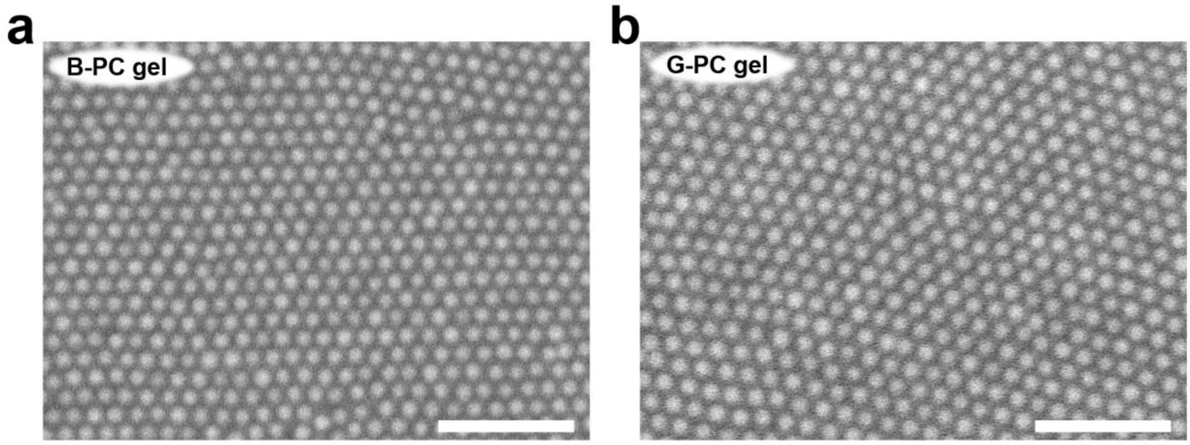

Supplementary Fig. 32: Physical microstructure characterization of multimode PC gels

The SEM images of **a** B-PC gel and **b** G-PC gel prepared by 220 nm and 255 nm diameter of RTP SiO_2 NPs, respectively (scale bar: 2 μm).

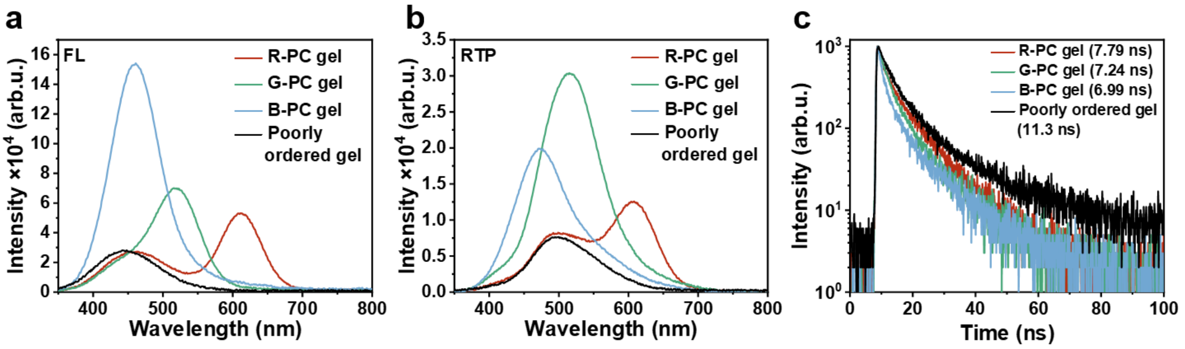

Supplementary Fig. 33: The influence of PC gels structure on FL on RTP

a FL and **b** RTP spectra of different gels under 365 nm light excitation, θ_1 and θ_2 is 90°. **c** Photoluminescence lifetime curves of different gels excited by a 375 nm laser and monitored at 466 nm wavelength. Source data are provided as a Source Data file.

Discussion: The FL and RTP spectra for B-, G-, and R-PC gels, as well as disordered gel, are measured under identical conditions. As shown in the Supplementary Fig. 33a and b, FL exhibits the strongest intensity in the B-PC gel, while RTP shows the strongest intensity in the G-PC gel. We measure the photoluminescence (PL) lifetimes of different gels, and the results show that the B-PC gel has the shortest PL lifetime (Supplementary Fig. 33c). This phenomenon is related to the resonant coupling effect of the PCs and the enhancement of the local density of states (LDOS).[4-7] Specifically, when the emission bands of FL and RTP align with the PBG of the PCs, the excited photons localize in the micro-nano structure of the PCs, enhancing the resonance efficiency between the emitted light and the photonic structure. As a result, the photon radiation recombination rate increases, thereby enhancing the emission intensity. Besides, the strong RTP emission in G-PC gel suggests that the resonance interaction between the emission light and photonic structure is independent of the type of molecular transition, whether S_1-S_0 or T_1-S_0.

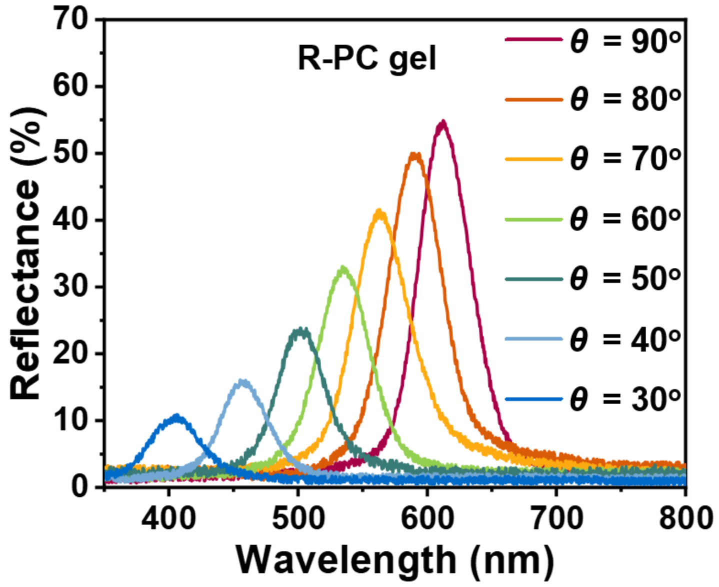

Supplementary Fig. 34: Angle-dependent PBGs of R-PC gel

Angle-dependent reflection spectra of the R-PC gel self-assembled by RTP SiO_2 NPs with diameter of 284 nm. Source data are provided as a Source Data file.

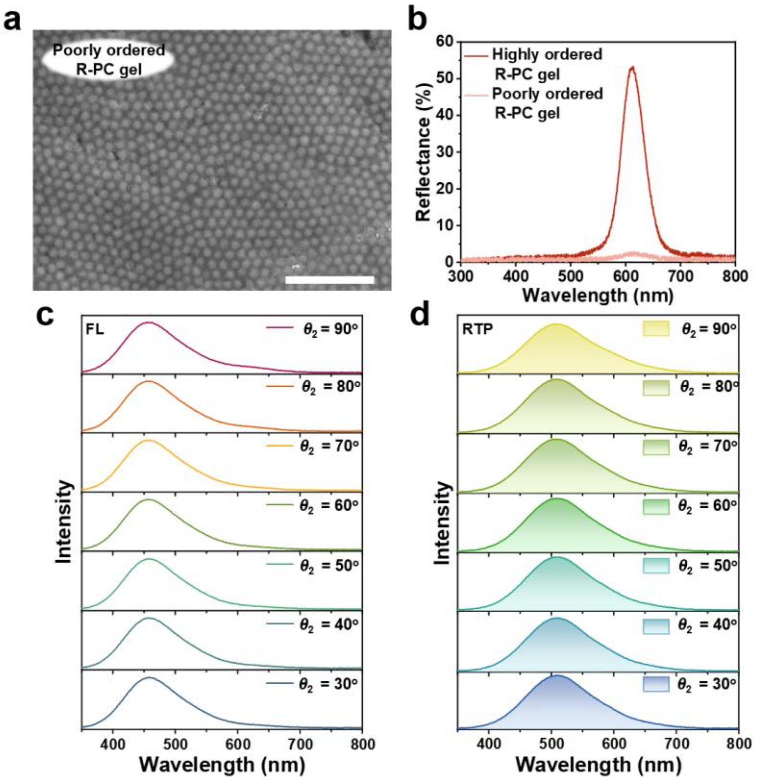

Supplementary Fig. 35: Multimode angle-dependent chromatic properties of disordered micro-structured R-PC gel

a The SEM images of poorly ordered R-PC gel prepared by 284 nm RTP SiO_2 NPs (scale bar: 2 μm). **b** The reflection spectra of highly ordered and poorly ordered R-PC gel, θ_1=90º and θ_2=90º. **c** and **d** Angle-dependent FL and RTP spectra of poorly ordered R-PC gel. Source data are provided as a Source Data file.

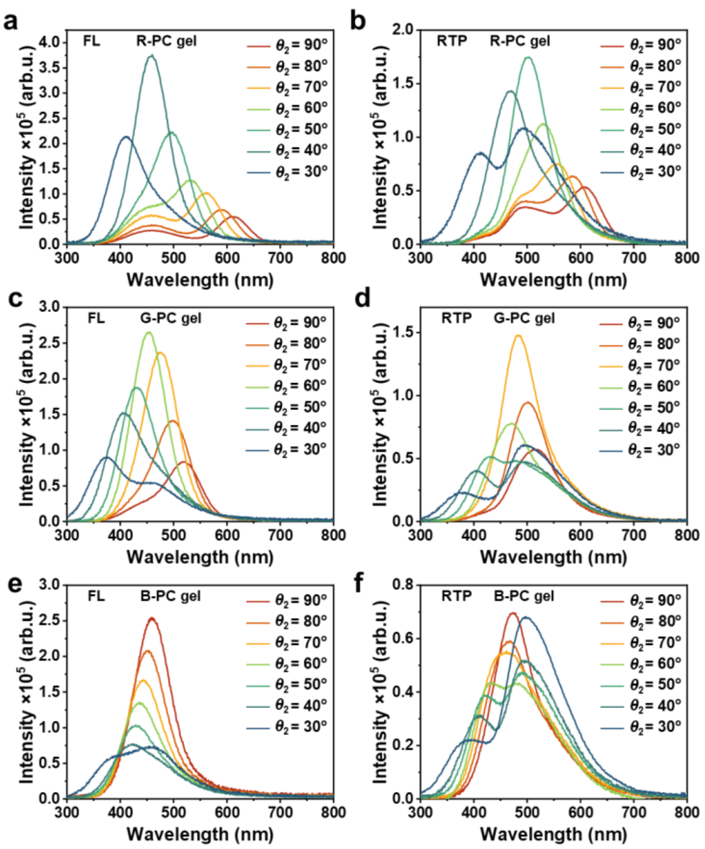

Supplementary Fig. 36: FL and RTP angle-dependent characteristics of PC gels

a and **b** Angle-dependent FL and RTP spectra of R-PC gel. **c** and **d** Angle-dependent FL and RTP spectra of G-PC gel. **e** and **f** Angle-dependent FL and RTP spectra of B-PC gel. Source data are provided as a Source Data file.

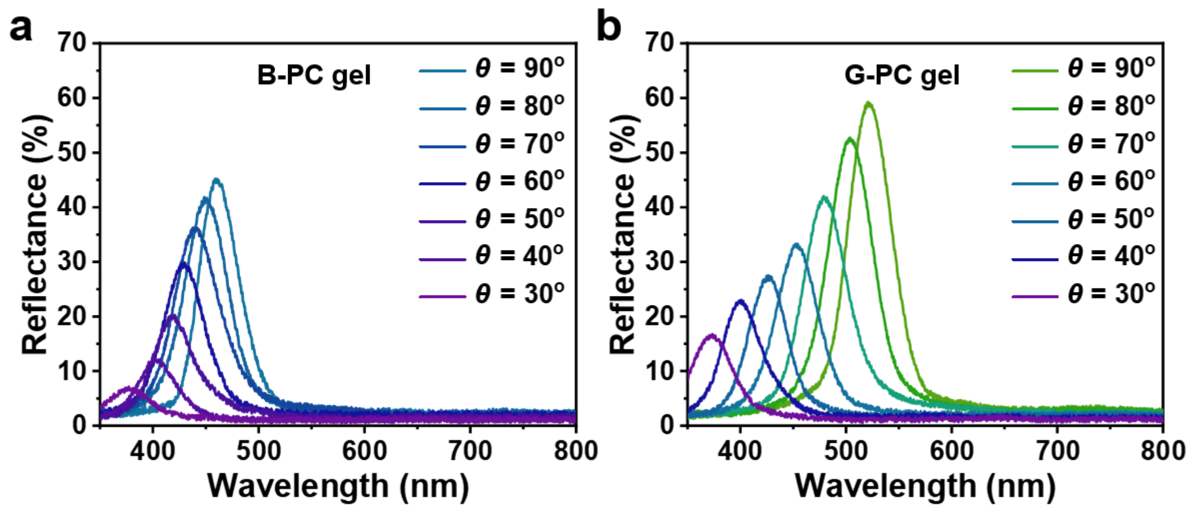

Supplementary Fig. 37: Angle-dependent PBGs of B-PC gel and G-PC gel

Angle-dependent reflection spectra of **a** B-PC gel and **b** G-PC gel self-assembled by RTP SiO_2 NPs with diameter of 220 nm and 239 nm, respectively. Source data are provided as a Source Data file.

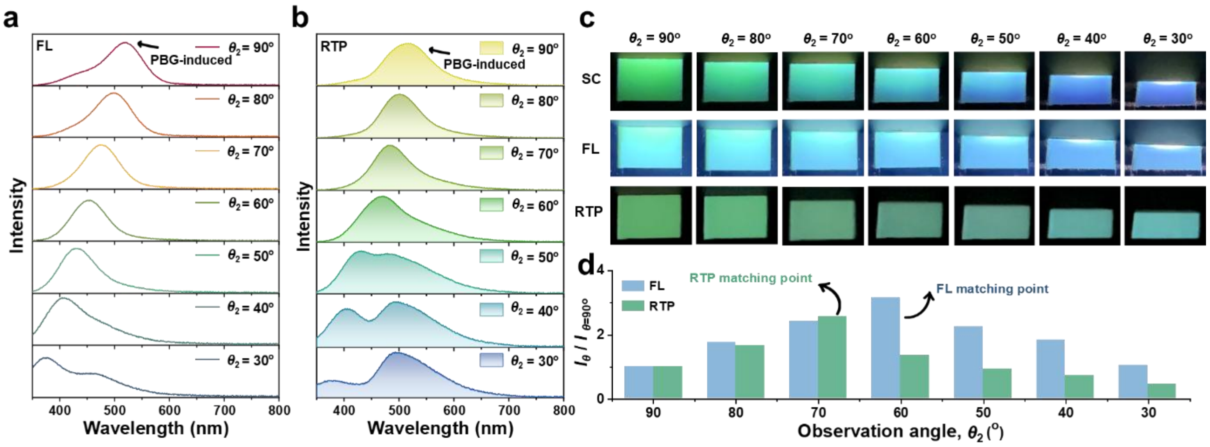

Supplementary Fig. 38: Multimode angle-dependent chromatic properties of G-PC gel

a and **b** Angle-dependent FL and RTP spectra of G-PC gel. **c** The photographs of the angle-dependent SC, FL and RTP of the G-PC gel under daylight, 365 nm UV lamp on and off, respectively. **d** The relationship between the intensities of FL and RTP of G-PC gel and the observed angle, where I_θ is the intensity at different θ_2 and $I_{\theta=90^\circ}$ is the intensity at θ_2=90°. Source data are provided as a Source Data file.

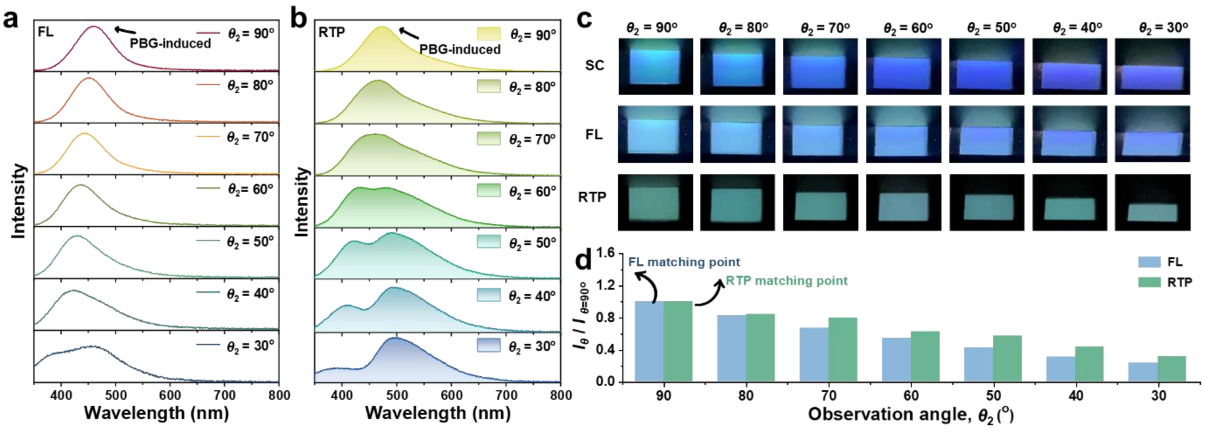

Supplementary Fig. 39: Multimode angle-dependent chromatic properties of B-PC gel

a and **b** Angle-dependent FL and RTP spectra of B-PC gel. **c** The photographs of the angle-dependent SC, FL and RTP of the B-PC gel under daylight, 365 nm UV lamp on and off, respectively. **d** The relationship between the intensities of FL and RTP of B-PC gel and the observed angle, where I_θ is the intensity at different θ_2 and $I_{\theta=90^\circ}$ is the intensity at θ_2=90°. Source data are provided as a Source Data file.

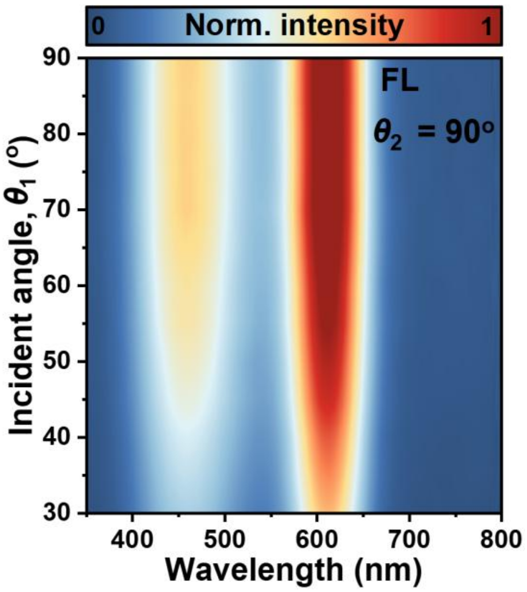

Supplementary Fig. 40: Optical behavior of the incident angle of excitation light on R-PC gel

Scanning θ_1-dependent two-dimensional FL spectra of R-PC gel. Source data are provided as a Source Data file.

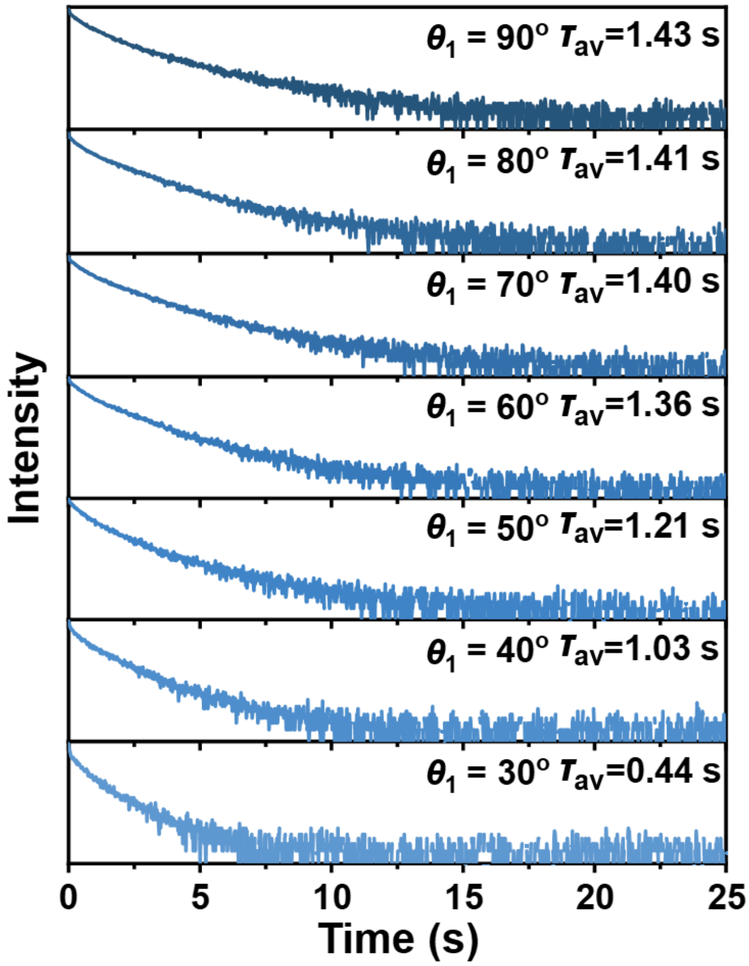

Supplementary Fig. 41: Variation of RTP lifetime on the incident angle of excitation light

RTP lifetime decay curves measured at different θ_1. Source data are provided as a Source Data file.

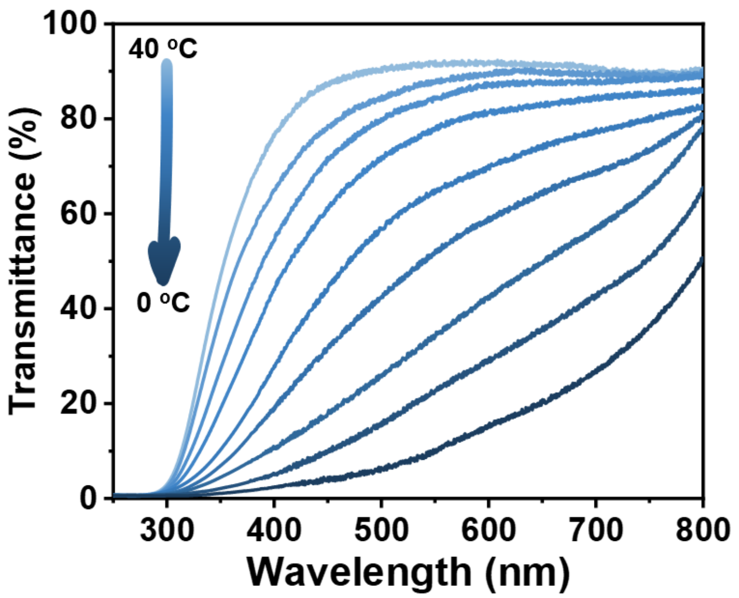

Supplementary Fig. 42: Thermal transition properties of transparent states and scattering states of R-PC gel

Temperature-dependent transmission spectra of R-PC gel. Source data are provided as a Source Data file.

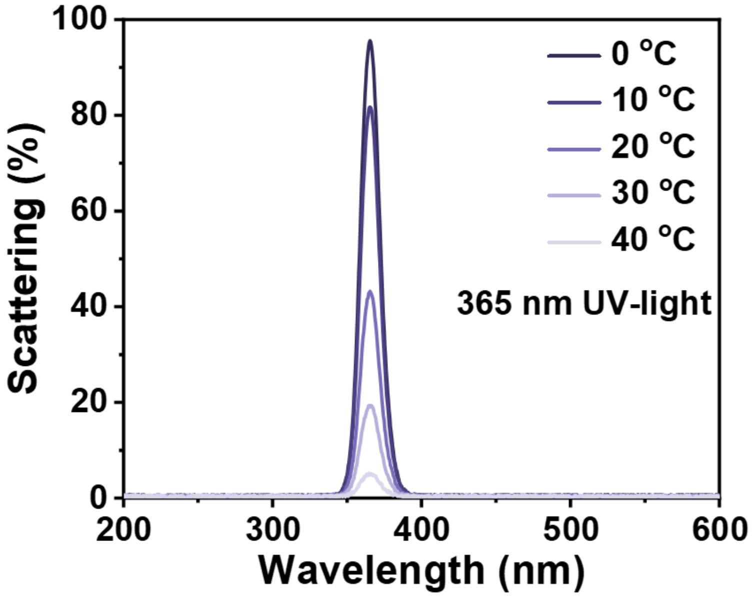

Supplementary Fig. 43: Enhanced behavior of UV excited light in the scattered state

Scattering spectra of excitation light incident on the R-PC gel at different temperature. Source data are provided as a Source Data file.

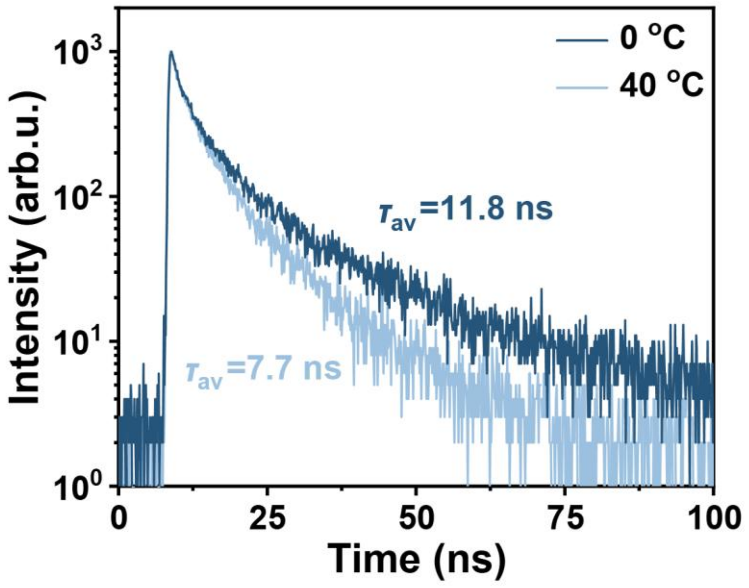

Supplementary Fig. 44: FL Lifetime of R-PC gel in refractive index matched state and mismatched state

The FL lifetime decay spectra of R-PC gel at 0 and 40 °C, respectively. Source data are provided as a Source Data file.

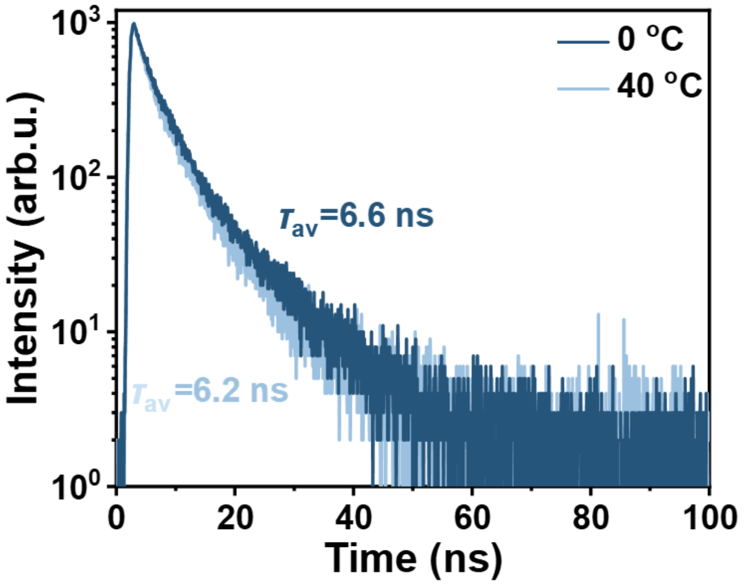

Supplementary Fig. 45: The inherent temperature-dependent photoluminescence properties of RTP SiO_2 NPs

The FL decay spectra of RTP SiO_2 NPs at 0 and 40 ºC, respectively. Source data are provided as a Source Data file.

Discussion: To verify that the increase in lifetime shown in Supplementary Fig. 43 is independent of the intrinsic temperature-dependent optical properties of RTP SiO_2 NPs, We have measured the intrinsic temperature-dependent FL lifetime of RTP SiO_2 NPs and found that compared to the significant increase in lifetime caused by self-scattering, the intrinsic temperature-dependent FL lifetime remained almost unchanged within the temperature range of 0 to 40 °C (Supplementary Fig. 44), thus the intrinsic temperature-dependent FL lifetime of RTP SiO_2 NPs is excluded.

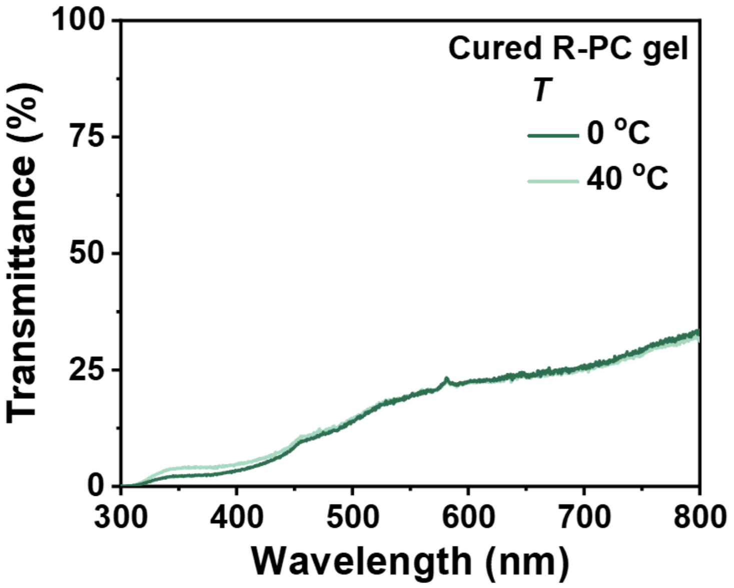

Supplementary Fig. 46: The temperature-dependent transmittance characteristics of the cured R-PC gel

Transmission spectra of cured R-PC gel with photo-initiators under different temperature. Source data are provided as a Source Data file.

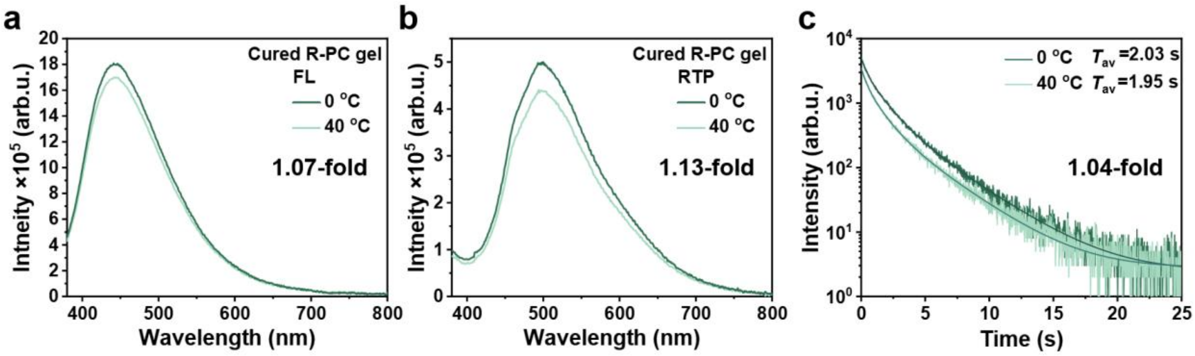

Supplementary Fig. 47: The inherent temperature effects of FL and RTP of R-PC gel

a FL, **b** RTP and **c** RTP decay spectra of cured R-PC gel under different temperature. Source data are provided as a Source Data file.

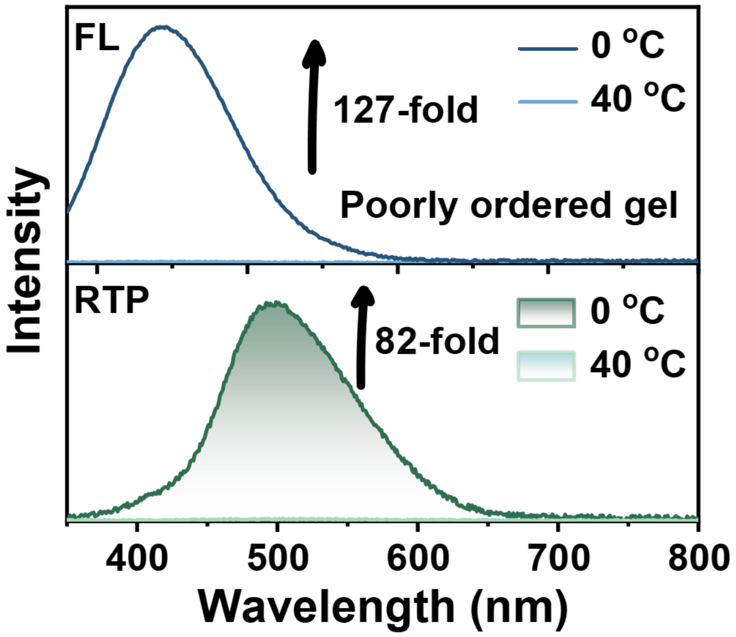

Supplementary Fig. 48: Scattering enhancement behavior of poorly ordered PC gel

The FL and RTP spectra of poorly ordered PC gel at 0 and 40 °C, respectively. Source data are provided as a Source Data file.

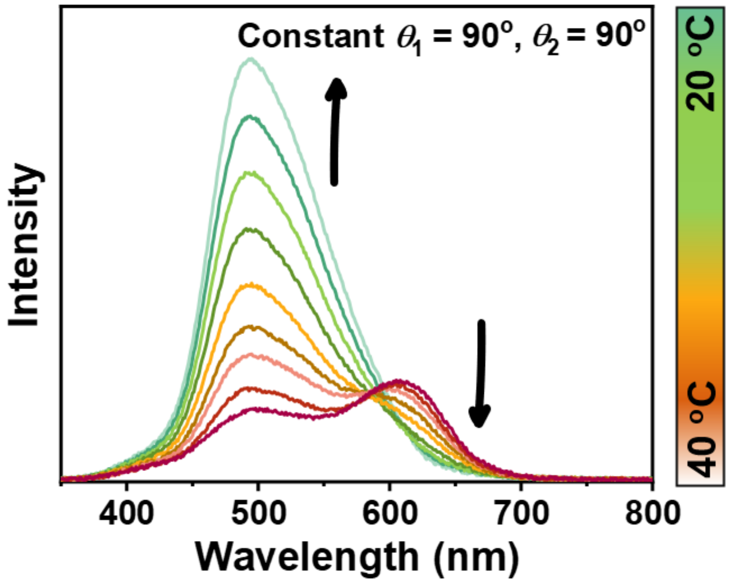

Supplementary Fig. 49: RTP thermochromic properties of R-PC gel

Temperature-dependent RTP spectra of R-PC gel, θ_1=90° and θ_2=90°. Source data are provided as a Source Data file.

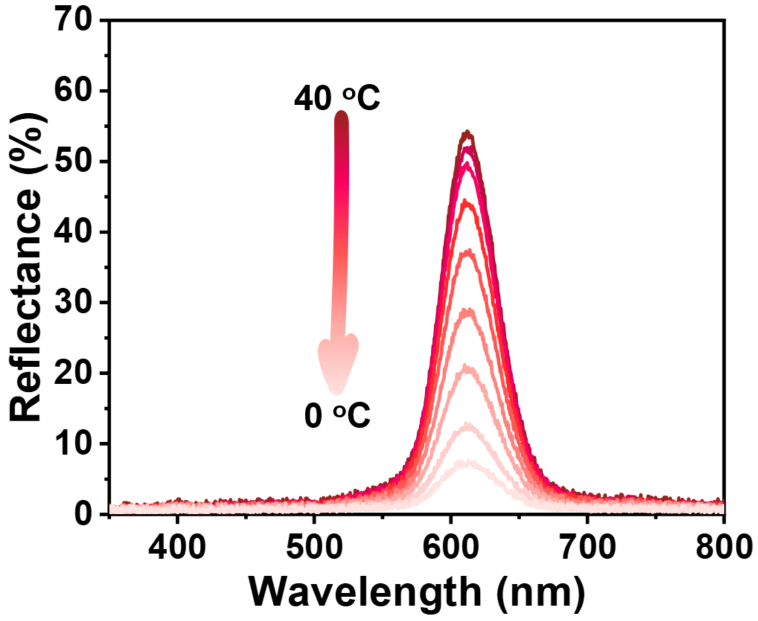

Supplementary Fig. 50: Scattering enhancement at low temperatures leads to PBG destruction

Temperature-dependent calibrated relative reflection spectra of R-PC gel, θ_1=90° and θ_2=90°. Source data are provided as a Source Data file.

Supplementary Table 1: Summary of RTP lifetimes of the RTP SiO_2 NPs obtained at different temperatures (77 -347 K), all quantitative measurements are performed in five replications (*n*=5) to calculate 95% confidence intervals.

T (K)	τ_1 (s)	A_1	τ_2 (s)	A_2	τ_3 (s)	A_3	τ_{av} (s)
77	1.328±0.045	3720±111	1.278±0.065	5219±132	11.591±0.484	420±33	4.378±0.362
107	1.206±0.063	3141±84	1.555±0.050	1239±76	8.147±0.701	377±11	3.760±0.349
137	1.079±0.057	3152±47	2.827±0.058	1175±52	7.221±0.386	355±11	3.404±0.144
167	0.804±0.035	4255±153	3.030±0.045	1726±64	7.081±0.336	314±8	3.162±0.116
197	0.852±0.022	4730±92	3.108±0.104	2429±102	6.710±0.494	335±31	3.033±0.172
227	0.582±0.026	4405±50	2.506±0.129	1637±68	5.931±0.429	370±9	2.731±0.235
257	0.753±0.053	4935±56	2.739±0.044	2715±58	5.216±0.383	329±6	2.498±0.098
287	0.726±0.047	3237±96	2.457±0.118	1992±68	4.230±0.331	313±5	2.258±0.114
317	0.583±0.043	1318±29	1.937±0.081	1412±78	3.120±0.227	260±6	1.920±0.097
347	0.475±0.024	1167±44	1.710±0.073	1497±43	2.806±0.233	241±7	1.728±0.070

Supplementary Table 2: Summary of RTP lifetimes of the RTP SiO_2 NPs obtained at different calcination temperatures, all quantitative measurements are performed in five replications (n=5) to calculate 95% confidence intervals.

T (ºC)	τ_1 (s)	A_1	τ_2 (s)	A_2	τ_3 (s)	A_3	τ_{av} (s)
325	0.247± 0.054	246± 191	0.035± 0.009	499± 13	1.219± 0.060	24± 1.6	0.473± 0.029
375	0.181± 0.064	285± 12	0.377± 0.011	208± 8	1.298± 0.036	118± 9	0.843± 0.023
425	0.162± 0.031	1512± 65	0.734± 0.026	824± 39	2.008± 0.078	190± 11	1.041± 0.091
475	0.572± 0.026	1396± 68	1.159± 0.115	1611± 67	2.976± 0.11	309± 4	1.499± 0.030
525	2.283± 0.052	1781± 54	1.286± 0.030	1838± 57	3.323± 0.084	351± 11	1.753± 0.066
575	0.660± 0.053	2337± 61	1.977± 0.068	1845± 57	3.856± 0.112	317± 14	2.021± 0.036
625	0.445± 0.040	1494± 51	1.573± 0.043	1411± 33	3.053± 0.045	315± 19	1.749± 0.039
675	0.137± 0.034	1232± 111	0.631± 0.026	1296± 116	1.889± 0.088	433± 16	1.156± 0.073
725	0.362± 0.041	602± 35	0.102± 0.010	473± 21	1.339± 0.078	153± 9	0.773± 0.053
775	0.207± 0.051	294± 15	0.073± 0.004	339± 17	1.191± 0.051	58± 2	0.631± 0.031
825	0.303± 0.048	284± 18	0.223± 0.016	224± 21	0.831± 0.079	103± 4	0.491± 0.043

Supplementary Table 3: Summary of the PLQYs of RTP SiO_2 NPs obtained at different calcination temperatures, all quantitative measurements are performed in five replications (*n*=5) to calculate 95% confidence intervals.

T (°C)	PLQY (%)
325	6.10±0.29
375	8.40±0.17
425	9.45±0.31
475	11.29±0.55
525	15.76±0.21
575	18.13±0.60
625	15.63±0.34
675	11.32±0.26
725	10.89±0.74
775	10.05±0.53
825	7.50±0.58

Supplementary Table 4: The mass and yield of RTP SiO_2 NPs prepared at different scales.

Magnification factor	Precursor solution (mL)				Mass (g)	yield
	Ethanol	Ammonia	Glucose solution	TEOS/Ethanol		
1-fold	130	16	6	12/10	2.9± 0.03	100%
25-fold	3250	400	150	300/250	72.1± 0.93	≈99.4%
250-fold	32500	4000	1500	3000/2500	719.4± 2.08	≈99.2%

Supplementary References

1. Varshni, Y. P. Temperature dependence of the energy gap in semiconductors. *Physica* **34**, 149-154 (1967). https://doi.org/10.1016/0031-8914(67)90062-6
2. Yu, P. et al. Temperature-Dependent Fluorescence in Carbon Dots. *J. Phys. Chem. C* **116**, 25552-25557 (2012). https://doi.org/10.1021/jp307308z
3. Zhu, S. et al. Photoluminescence mechanism in graphene quantum dots: Quantum confinement effect and surface/edge state. *Nano Today* **13**, 10-14 (2017). https://doi.org/10.1016/j.nantod.2016.12.006
4. Chen, X. et al. Dynamic regulation of photoluminescence based on mechanochromic photonic elastomers. *Chem. Eng. J.* **426**, 131259 (2021). https://doi.org/10.1016/j.cej.2021.131259
5. Ganesh, N. et al. Enhanced fluorescence emission from quantum dots on a photonic crystal surface. *Nature Nanotech.* **2**, 515-520 (2007). https://doi.org/10.1038/nnano.2007.216
6. Yuan, S. et al. et al. Fluorescence enhancement of perovskite nanocrystals using photonic crystals. *J. Mater. Chem. C* **9**, 908-915 (2021). https://doi.org/10.1039/D0TC05056C
7. Lee, H. et al. Structurally engineered colloidal quantum dot phosphor using TiO_2 photonic crystal backbone. *Light: Sci. Appl.* **11**, 318 (2022). https://doi.org/10.1038/s41377-022-01020-2
