# Supplementary material for: Scalable synthesis of phosphorescent SiO2 nanospheres and their use for angle-dependent and thermoresponsive photonic gels with multimode luminescence
Source: Nat Commun. 2025 Jul 18;16:6640. doi: 10.1038/s41467-025-61967-9 (PMC12274461; doi:10.1038/s41467-025-61967-9)
Supplement: Supplementary file 2 — Description of Additional Supplementary Files [file 41467_2025_61967_MOESM2_ESM.pdf]

Description of Additional Supplementary Files

File Name: Supplementary Movie 1

Description: The behavior of color afterglow of RTP SiO_2 NPs under different excitation light.

File Name: Supplementary Movie 2

Description: Demonstration of angle-dependent structural color, fluorescence and time-dependent afterglow of self-assembled multimode PCs.

File Name: Supplementary Movie 3

Description: The process of angle-dependent chromatic behavior of R-PC gel.

File Name: Supplementary Movie 4

Description: The process of stretching-induced transition from transparent state to the scattering state, and self-scattering FL enhancement behavior of R-PC gel.

File Name: Supplementary Movie 5

Description: Demonstration of RTP properties of PC gel in the matched and unmatched state of refractive index.
